# Diagnostic accuracy and added value of blood-based protein biomarkers for pancreatic cancer: A meta-analysis of aggregate and individual participant data

**DOI:** 10.1016/j.eclinm.2022.101747

**Published:** 2022-11-24

**Authors:** Lenka N.C. Boyd, Mahsoem Ali, Mariska M.G. Leeflang, Giorgio Treglia, Ralph de Vries, Tessa Y.S. Le Large, Marc G. Besselink, Elisa Giovannetti, Hanneke W.M. van Laarhoven, Geert Kazemier

**Affiliations:** a Amsterdam UMC, Location Vrije Universiteit, Department of Surgery, Amsterdam, the Netherlands; b Amsterdam UMC, Location Vrije Universiteit, Department of Medical Oncology, Lab of Medical Oncology, Amsterdam, the Netherlands; c Cancer Center Amsterdam, Imaging and Biomarkers, Amsterdam, the Netherlands; d Amsterdam UMC, Location University of Amsterdam, Department of Epidemiology and Data Science, Amsterdam, the Netherlands; e Clinic of Nuclear Medicine, Imaging Institute of Southern Switzerland, Ente Ospedaliero Cantonale, Bellinzona, Switzerland; f Department of Nuclear Medicine and Molecular Imaging, Lausanne University Hospital, Lausanne, Switzerland; g Faculty of Biology and Medicine, Università Della Svizzera Italiana, Lugano, Switzerland; h Amsterdam UMC, Location Vrije Universiteit, Medical Library, Amsterdam, the Netherlands; i Department of Surgery, Dijklander Ziekenhuis Location Hoorn, Hoorn, the Netherlands; j Amsterdam UMC, Location University of Amsterdam, Department of Surgery, Amsterdam, the Netherlands; k Cancer Pharmacology Lab, AIRC Start-Up Unit, Fondazione Pisana per la Scienza, Pisa, Italy; l Amsterdam UMC, Location University of Amsterdam, Department of Medical Oncology, Amsterdam, the Netherlands

## Abstract

**Background:**

Novel blood-based protein biomarkers may be of value for efficient, accurate, and non-invasive diagnosis of pancreatic cancer. This study assesses the diagnostic accuracy of newly recognized blood-based protein biomarkers for detecting pancreatic cancer, and investigates their added value to CA19-9, the common blood-based biomarker in clinical use for pancreatic cancer.

**Methods:**

PubMed, Embase, Web of Science, and the Wiley/Cochrane Library were systematically searched from inception until June 2022. A meta-analysis of aggregate and individual participant data was conducted using frequentist and Bayesian hierarchical random-effects models. The added clinical utility of protein biomarkers was investigated using bootstrap bias-corrected decision curve analyses.

**Findings:**

Aggregate data from 28 primary studies (6127 participants) were included, of which 8 studies (1790 participants) provided individual participant data. CA19-9 was significantly more accurate than MIC-1 for distinguishing pancreatic cancer from benign disease (AUC, 0.83 *vs* 0.74; relative diagnostic odds ratio [rDOR], 2.10 [95% CI, 0.98–4.48]; p = 0.002), THBS2 (AUC, 0.87 *vs* 0.69; rDOR, 4.53 [2.16–9.39]; p < 0.0001), TIMP-1 (AUC, 0.91 *vs* 0.70; rDOR, 8.00 [3.81–16.9]; p < 0.0001), OPN (AUC, 0.89 *vs* 0.74; rDOR, 4.22 [1.13–15.6]; p < 0.0001), ICAM-1 (AUC, 0.91 *vs* 0.68; rDOR 9.30 [0.87–99.5]; p < 0.0001), and IGFBP2 (AUC, 0.91 *vs* 0.68; rDOR, 4.48 [0.78–24.3]; p < 0.0001). The addition of these novel protein biomarkers to CA19-9 did not significantly improve the AUC, and resulted in minor increases or limited decreases in clinical utility.

**Interpretation:**

Novel protein biomarkers have moderate diagnostic accuracy, do not outperform CA19-9 in differentiating pancreatic cancer from benign disease, and show limited added clinical value to CA19-9. We propose recommendations to aid the development of minimally invasive diagnostic tests with sufficient clinical utility to improve the management of patients with suspected pancreatic cancer.

**Funding:**

Bennink Foundation, Dutch Cancer Foundation (KWF Kankerbestrijding), and AIRC.

**Research in context**

**Evidence before this study**

Pancreatic ductal adenocarcinoma (PDAC) is an aggressive disease with an overall poor prognosis and poses a major diagnostic challenge due to a lack of accurate biomarkers. Carbohydrate antigen 19-9 (CA19-9) is currently the main blood-based biomarker in clinical use for pancreatic cancer, but has a very low diagnostic power. Although numerous protein biomarkers for diagnosing PDAC have been discovered in recent years, it is unclear whether these proteins outperform CA19-9 or have added clinical value to CA19-9. To investigate the diagnostic accuracy and added value of these proteins, we conducted systematic literature searches in the bibliographic databases Medline, Embase, Wiley/Cochrane Library, and Web of Science (Core Collection) from inception up to June 15, 2022, in collaboration with a medical information specialist. We included diagnostic accuracy studies that evaluated the diagnostic performance of blood-based protein biomarkers for PDAC, and we requested individual participant data of all included studies.

**Added value of this study**

To date, this meta-analysis is the most comprehensive in evaluating the diagnostic accuracy of novel blood-based proteins to detect PDAC, and their added clinical utility to CA19-9. Our findings indicate that novel protein biomarkers show poor or moderate diagnostic accuracy, and do not outperform CA19-9 in distinguishing PDAC from benign disease. In addition, the added clinical value of novel protein biomarkers to CA19-9 is either limited or unknown.

**Implications of all the available evidence**

This study shows that currently recognized protein biomarkers are not sufficiently accurate to be prospectively validated or clinically implemented. Novel biomarkers and prediction models should demonstrate sufficient added value over CA19-9 in terms of diagnostic accuracy and clinical utility, before validation or implementation. We propose recommendations for future studies investigating diagnostic blood-based protein biomarkers for PDAC. These will help to improve blood-based diagnostic biomarker research practice, stimulate efficient use of laboratory resources, and prevent unnecessary diagnostic testing of patients.

## Introduction

Pancreatic ductal adenocarcinoma (PDAC) poses a major diagnostic and therapeutic challenge as symptoms often develop in an advanced disease stage and these patients typically are diagnosed too late.^1^ Consequently, the prognosis for patients with PDAC is poor, with a dismal relative 5-year survival rate of 10.8%.^2^ The opportunity for improving this prognosis lies foremost in accurate and early disease detection, which could aid earlier treatment initiation, and increase the possibility of surgical intervention.^3^

The modalities currently used for diagnosing PDAC are a combination of clinical symptoms combined with radiological findings, and pathological confirmation of the diagnosis by fine-needle aspiration or brush cytology. These diagnostic measures are invasive and time-consuming – and often the diagnosis remains inconclusive until after surgery is performed.^3^ Carbohydrate antigen 19-9 (CA19-9) is a protein most commonly used as a non-invasive tumor biomarker for patients suspected of PDAC. CA19-9 is produced in normal pancreas tissue, but can be highly expressed by cancerous pancreatic cells and is therefore regarded as a tumor marker that can support the diagnosis of PDAC. However, as its diagnostic power is perceived to be limited – with a reported sensitivity and specificity between 82% and 91% – it is mainly used for monitoring disease and follow-up after therapy.^4^ Additionally, Lewis-negative patients are not able to produce CA19-9, which hampers its diagnostic utility. As such, an efficient, accurate, and minimally invasive method for diagnosing PDAC remains an unmet medical need.

In recent years, many potential blood-based diagnostic biomarkers have been investigated, such as circulating tumor cells and DNA, or microRNAs. However, none of these potential biomarkers have reached the phase of validation and implementation in the clinic. The addition of protein biomarkers to CA19-9 could offer a feasible and easily implementable diagnostic aid.^5^^,^^6^ These blood-based biomarkers would likely not replace but complement the current diagnostic pipeline at the onset of presentation of a patient, and could potentially save time and prevent invasive procedures.

Several studies have focused on detection of blood proteins or protein panels which may be of diagnostic value for patients with PDAC. A systematic review and meta-analysis of blood-based diagnostic proteins in comparison with and in addition to CA19-9 is currently lacking. This systematic review and meta-analysis evaluates the pooled diagnostic accuracy of blood-based proteins to detect PDAC, and their incremental clinical utility over CA19-9.

## Methods

This systematic review and meta-analysis is reported in accordance with the Preferred Reporting Items for Systematic Review and Meta-Analysis of Diagnostic Test Accuracy Studies Statement.^7^^,^^8^

### Search strategy

Systematic literature searches were conducted in the bibliographic databases Medline, Embase, Wiley/Cochrane Library and Web of Science (Core Collection) from inception up to November 2, 2021, in collaboration with a medical information specialist. An updated search was conducted on June 15, 2022. Reference lists of primary studies and review articles were screened for additional publications. The full search strategies for all databases can be found in the Supplementary Appendix.

### Eligibility criteria

Articles were included that collected blood samples at the time of diagnosis and reported the diagnostic accuracy of blood-based protein biomarkers for PDAC in adults. Studies were excluded that collected blood samples after chemotherapy, included less than ten patients in either the PDAC or the control group, did not use histopathological diagnosis as the reference standard for diagnosing PDAC, or did not provide enough data to construct two-by-two diagnostic contingency tables. For comparative studies between CA19-9 and blood-based proteins, articles were included that used a fully paired or randomized design.

### Study selection and screening

Two reviewers (L.N.C.B. and M.A.) independently screened all potentially relevant titles and abstracts for eligibility. The full text of potentially eligible articles was checked for the inclusion criteria (Supplementary Table S1), and differences in judgement were resolved through a consensus procedure. Corresponding authors of all included studies were contacted three times over the course of a month to request individual participant data (IPD) and to inquire whether any unpublished data regarding diagnostic protein biomarkers for PDAC were available. IPD data of eight studies were obtained, the data of five other studies were not available anymore, six studies did not have the requested information readily available, and authors of nine studies did not respond.

### Data extraction and quality assessment

Two authors (L.N.C.B. and M.A.) independently extracted data from primary studies using piloted forms. A modified Quality Assessment of Diagnostic Accuracy Studies 2 (QUADAS-2) tool was used for diagnostic test accuracy studies and the QUADAS-C tool for comparative designs.^9^^,^^10^ These tools were adapted to incorporate additional signaling questions regarding the use of data-driven selection of optimal cutoffs, timing of blood collection, and choice of reference standards.

### Data synthesis and statistical analysis

To account for expected between-study variability in reported thresholds and jointly synthesize sensitivity and specificity, the diagnostic test accuracy of each biomarker was summarized across studies using a generalized nonlinear mixed modeling approach, ie, with the Rutter and Gatsonis hierarchical summary receiver operating characteristic (HSROC) model.^11^ Due to a low number of studies, a symmetric HSROC model (ie, without the shape parameter) was fitted to facilitate convergence of the model.

Direct comparisons between index tests, eg, MIC-1 and CA19-9, were performed by including test type as a covariate to the symmetric HSROC model and calculating the diagnostic odds ratio (DOR) of each index test. The DOR is a summary measure of diagnostic accuracy and describes how many times higher the odds of a positive test are for a patient with PDAC than for a patient without PDAC. Non-informative tests have a DOR close to 1, while highly accurate tests have a DOR considerably higher than 1. For comparisons between single blood-based proteins and CA19-9, the relative diagnostic odds ratio (rDOR) is the ratio of the biomarkers’ respective DORs and describes the extent to which protein biomarkers outperform CA19-9. Therefore, an rDOR higher than 1 indicates a superior accuracy of protein biomarkers compared to CA19-9. Additionally, the diagnostic performance of protein biomarkers in distinguishing PDAC from healthy controls was compared with their accuracy in distinguishing PDAC from benign disease. In these meta-regression analyses, an rDOR lower than 1 indicates that the protein biomarker has a lower accuracy in PDAC *vs* benign disease compared with PDAC *vs* healthy controls. The area under the summary receiver operating characteristic (SROC) curve was estimated with numerical integration.

If frequentist HSROC models gave unreliable variance estimates of the accuracy parameter despite attempts at improving estimation of model parameters, a Bayesian symmetric HSROC model was fitted instead using Markov Chain Monte Carlo simulation, and 95% credible intervals (CrIs) were computed for parameters of interest. Prior distributions and other components of the modelling process (eg, the number of iterations and chains) were taken from version 2.0 of the Cochrane Handbook for Systematic Reviews of Diagnostic Test Accuracy.

For all HSROC meta-regression analyses, frequentist likelihood ratio tests were used to determine the statistical significance of removing covariates from the HSROC meta-regression model, if the model's convergence criterion was satisfied. If the variance of the accuracy parameter was estimated with high uncertainty despite convergence of the frequentist HSROC model, a likelihood ratio test was performed for statistical testing and a Bayesian HSROC model was used for estimation of model parameters.

Additionally, a pooled area under the curve (AUC) was estimated in a meta-analysis of the AUC of the primary studies, unless AUC estimates from fewer than three studies were available or very high between-study heterogeneity was observed. Specifically, the logit-transformed AUC from each study was used to estimate a pooled AUC in a random-effects meta-analysis with a restricted maximum likelihood estimator and Jackson's modification of the Hartung-Knapp-Sidik-Jonkman variance correction.^12^ If studies did not report confidence intervals, the standard error of the AUC was approximated with Newcombe's method.^13^

Heterogeneity in the HSROC analysis was assessed through visual inspection of SROC plots and was explored through meta-regression analyses. Between-study heterogeneity in the meta-analysis of AUC estimates was assessed using τ^2^ (reported on the logit scale), whereas the proportion of variability due to between-study heterogeneity was assessed using the I^2^ statistic.^14^ We used meta-regression analyses to identify possible sources of heterogeneity, and used an R^2^ statistic to study the potential importance of predictor variables (eg, serum *vs* blood, or the type of control group [healthy donors or benign disease]), as described previously.^15^ This R^2^ statistic was defined as the proportion of total between-study heterogeneity that can be explained by incorporating a predictor variable into the meta-regression model.

A two-stage IPD meta-analysis was performed to assess the added diagnostic value of protein biomarkers to CA19-9. In the first stage, the increase in AUC and corresponding confidence intervals were obtained using either the Hanley-McNeil method or using bootstrap resampling to account for the correlation between the AUCs of the two models.^16^^,^^17^ In the second stage, results were pooled across studies in a random-effects meta-analysis with a restricted maximum likelihood estimator and modified Hartung-Knapp-Sidik-Jonkman confidence intervals.

The added clinical value of protein biomarkers to CA19-9 was evaluated using the IPD data of primary studies. For each study, overoptimism-corrected decision curve analyses were performed using 1000 repeats of Harrell's bootstrap resampling procedure to compare the clinical utility of a prediction model using both CA19-9 and another protein biomarker with the clinical utility of a prediction model using CA19-9 only.^18^, ^19^, ^20^ At each decision threshold, the difference in standardized net benefit of the models was calculated to obtain the incremental clinical utility of the single protein biomarkers.

Two sets of sensitivity analyses were performed. First, for all eighteen HSROC meta-regression analyses, we restricted the analysis to comparative studies, which give unbiased estimates of relative accuracy.^10^^,^^21^ Second, for the decision curve analysis, we varied the prevalence of early-stage PDAC (ie, the pre-test probability), as pre-test probabilities cannot be estimated from multiple-gate (‘diagnostic case–control’) studies.

A formal statistical investigation of potential publication bias, ie, with Deeks’ test, was not performed, given the low power of funnel plot asymmetry tests to detect publication bias and small-study effects in the context of diagnostic test accuracy studies.^8^^,^^22^

A two-sided p-value of less than 0.05 was considered as statistically significant. All analyses were performed in R, version 4.2.1 (R Foundation for Statistical Computing), Stata, version 17.0 (StataCorp LLC), and SAS, version 9.4 (SAS Institute Inc.). Additional information regarding the statistical analyses is provided in the supplements.

### Role of the funding source

The funders had no role in the study design, data collection, analysis, interpretation, writing of the report, and in the decision to submit the paper for publication. L.N.C.B. and M.A. had full access to and verified the data in the study, and all authors had final responsibility for the decision to submit for publication.

## Results

### Search results

**Fig. 1:**
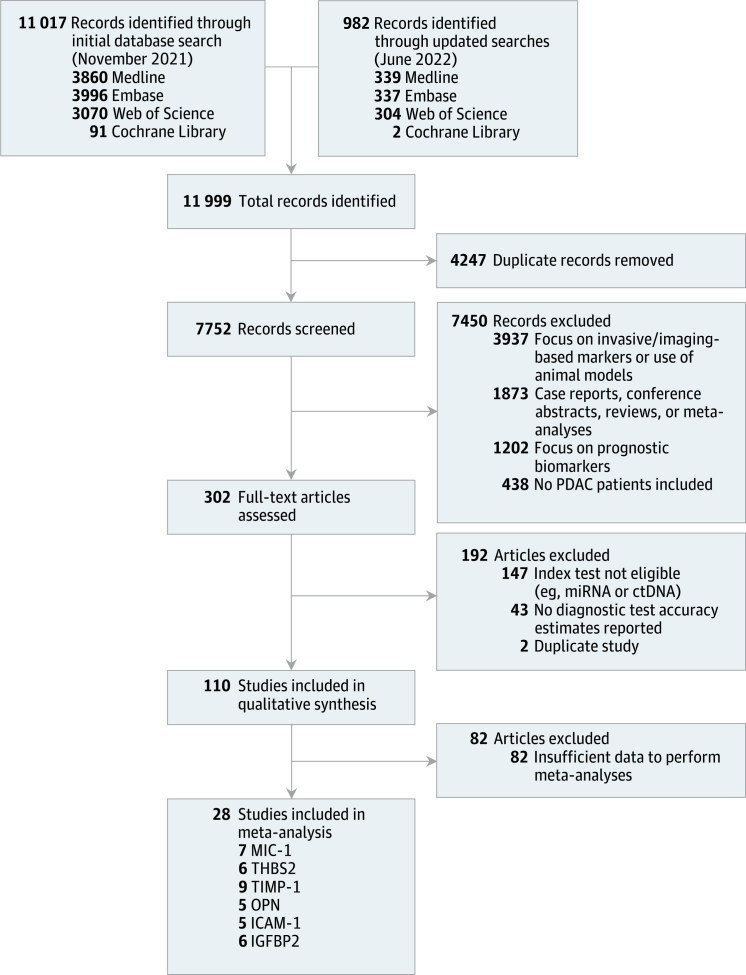
**Overviewof the study selection process.**

The literature search yielded 11,999 records, of which 7752 remained after removal of duplicates. Upon initial title and abstract screening, 7450 articles were excluded, and the remaining 302 articles were retained for full-text screening (Fig. 1). Of these, 28 studies met the eligibility criteria, reporting data on 6127 individuals (2770 patients with PDAC, 2082 healthy controls, and 1275 patients with benign disease).^23^, ^24^, ^25^, ^26^, ^27^, ^28^, ^29^, ^30^, ^31^, ^32^, ^33^, ^34^, ^35^, ^36^, ^37^, ^38^, ^39^, ^40^, ^41^, ^42^, ^43^, ^44^, ^45^, ^46^, ^47^, ^48^, ^49^, ^50^ Additionally, individual participant data from eight studies were obtained.^24^^,^^25^^,^^28^^,^^33^^,^^38^^,^^43^^,^^48^^,^^49^

### Study characteristics and risk of bias

The characteristics of all included studies are summarized in Supplementary Table S2. In total, 23 included studies (82%) assessed the diagnostic accuracy of blood-based proteins in 2579 patients with PDAC and 2082 healthy controls with a median of 62 patients with PDAC (IQR, 50–85) and a median of 50 healthy controls (IQR, 37–93) per study.^23^^,^^25^, ^26^, ^27^, ^28^, ^29^^,^^32^, ^33^, ^34^^,^^36^, ^37^, ^38^, ^39^, ^40^, ^41^, ^42^, ^43^^,^^45^, ^46^, ^47^, ^48^, ^49^, ^50^ The diagnostic test performance of protein biomarkers in PDAC *vs* benign disease was investigated in 21 studies (75%), comprising a total of 2229 patients with PDAC patients (median number per study, 62; IQR, 40–84) and 1275 patients with benign disease (median, 40; IQR, 24–86).^23^, ^24^, ^25^, ^26^^,^^29^, ^30^, ^31^, ^32^, ^33^, ^34^, ^35^^,^^37^^,^^38^^,^^40^^,^^42^, ^43^, ^44^, ^45^^,^^48^, ^49^, ^50^

The risk of bias of all included studies is summarized in Supplementary Fig. S1. All studies, except for the study of Byrling et al.^25^ and Hogendorf et al.,^30^ were rated at high or unclear risk of bias in the patient selection domain due to the use of multiple-gate (‘case–control’) designs or clinically unrepresentative control populations (ie, only healthy controls as the control group). Four studies (14%) were rated at high risk of bias in the index test domain^30^^,^^35^^,^^38^^,^^46^ because they used data-driven cut-off selection methods (eg, with the Youden index) to obtain optimal thresholds, which overestimate diagnostic performance estimates, especially in cases of low sample sizes.^51^ For an additional four studies it was unclear whether thresholds were pre-specified.^27^^,^^40^^,^^45^^,^^47^

In total, six proteins were analyzed in a sufficient number of studies to warrant their inclusion in the diagnostic meta-analysis: macrophage inhibitory cytokine 1 (MIC-1), thrombospondin-2 (THBS2), tissue inhibitor of metalloproteinase 1 (TIMP-1), osteopontin (OPN), intercellular adhesion molecule 1 (ICAM-1), and insulin-like growth factor-binding protein 2 (IGFBP2).

### MIC-1

**Fig. 2:**
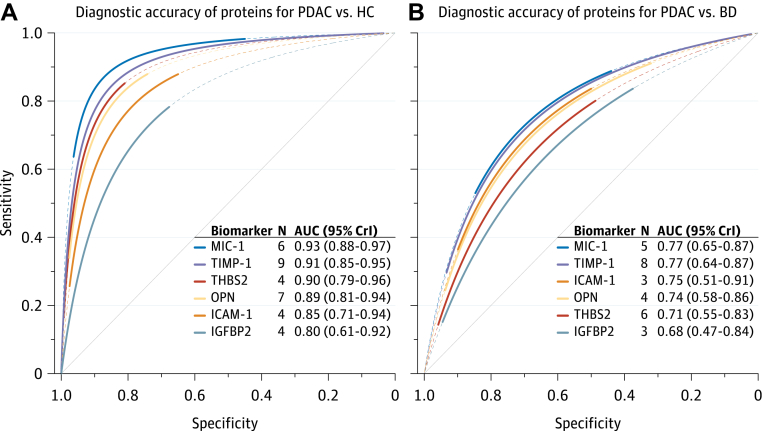
**SROC curves for PDAC *vs* healthy controls (A) and PDAC *vs* benign disease (B).** SROC curves are truncated at their respective maximal and minimal observed specificities. Extrapolation beyond these regions is indicated by the dashed lines. 95% CrI, 95% credible interval; AUC, area under the summary receiver operating characteristic (SROC) curve; BD, benign pancreatic diseases; HC, healthy controls; N, number of studies; PDAC, pancreatic ductal adenocarcinoma.

**Fig. 3:**
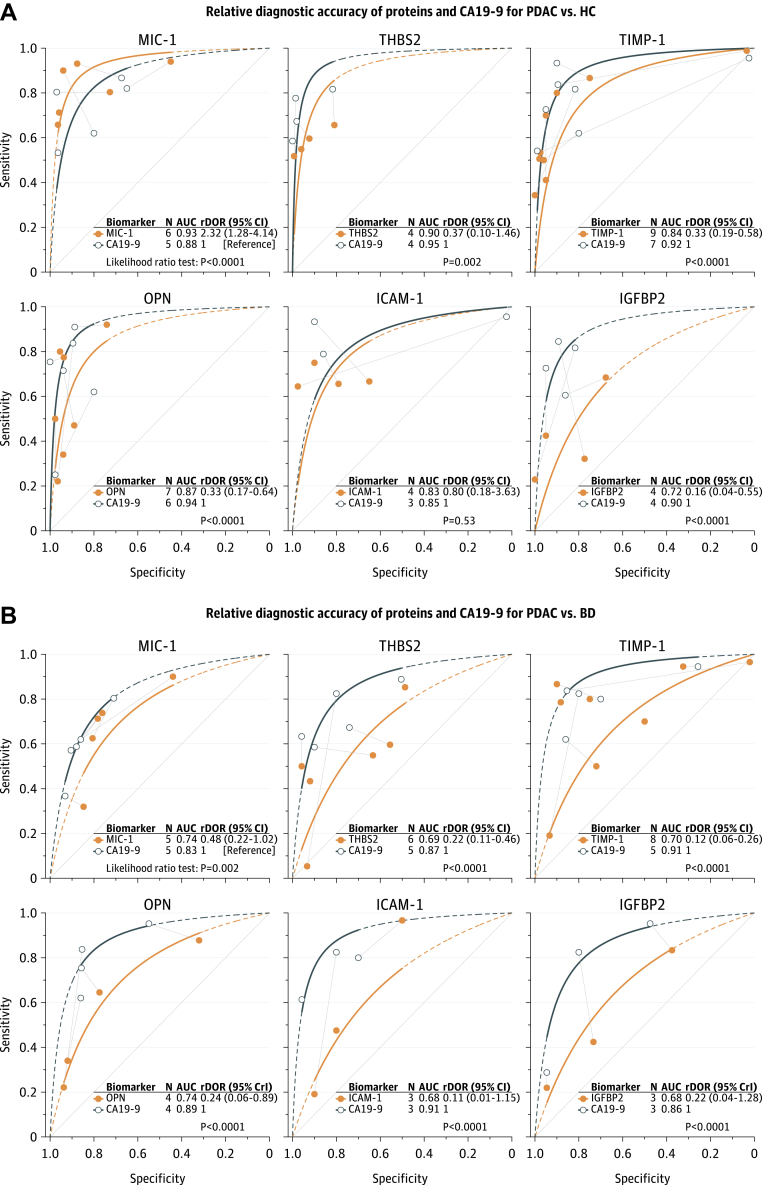
**Relative diagnostic accuracy of proteins and CA19-9 as diagnostic biomarkers for PDAC *vs* healthy controls (A) and PDAC *vs* benign disease (B).** Individual studies are presented as symbols, and head-to-head comparisons within studies are indicated with grey lines connecting two symbols. An rDOR lower than 1 indicates that the protein biomarker has a lower diagnostic accuracy than CA19-9. CrI, credible interval (derived from Bayesian hierarchical models); PDAC, pancreatic ductal adenocarcinoma.

**Fig. 4:**
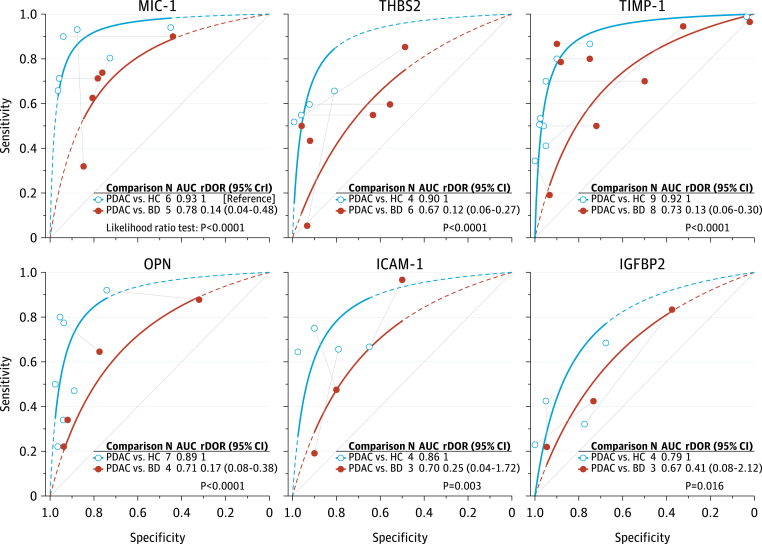
**Relative diagnostic accuracy of protein biomarkers for PDAC *vs* healthy controls and PDAC *vs* benign disease.** An rDOR lower than 1 indicates that the protein biomarker has a lower accuracy for pancreatic ductal adenocarcinoma *vs* benign disease compared with the protein's accuracy for pancreatic ductal adenocarcinoma *vs* healthy controls. CrI, credible interval (derived from Bayesian hierarchical models); PDAC, pancreatic ductal adenocarcinoma.

MIC-1 was the best performing protein in distinguishing PDAC from healthy controls (Fig. 2A and Supplementary Fig. S2A) with an area under the SROC curve (AUSROC) of 0.93 (95% CI 0.88–0.97). In HSROC meta-regression models, MIC-1 demonstrated higher accuracy than CA19-9 (rDOR 2.32 [95% CI 1.28–4.14]; p < 0.0001; Fig. 3A). However, there was strong evidence that MIC-1 was less accurate than CA19-9 for PDAC *vs* benign disease (rDOR 0.48 [95% CI 0.22–1.02]; p = 0.002; Fig. 3B). MIC-1 was substantially less accurate in distinguishing PDAC from benign disease than in distinguishing PDAC from healthy controls (rDOR 0.14 [95% CrI 0.04–0.48]; p < 0.0001; Fig. 4). The addition of MIC-1 to CA19-9 did not significantly improve the diagnostic accuracy for differentiating PDAC from benign disease (increase in AUC 0.07 [95% CI −0.07 to 0.21]; τ^2^ 0.00 [0.00–0.13]; I^2^ 73% [0–99%]; Supplementary Fig. S5A). However, the confidence interval for the increase in AUC was wide and was compatible with both large decreases and substantial increases in diagnostic accuracy.

### THBS2

THBS2 showed a lower diagnostic accuracy than CA19-9, both for PDAC *vs* healthy controls (rDOR 0.37 [95% CI 0.10–1.46]; p = 0.002) and for PDAC *vs* benign disease (rDOR 0.22 [95% CI 0.11–0.46]; p < 0.0001). Like MIC-1, THBS2 demonstrated lower accuracy in PDAC *vs* benign disease than in PDAC *vs* healthy controls (rDOR 0.12 [95% CI 0.06–0.27]; p < 0.0001). The combination of CA19-9 and THBS2 did not substantially improve diagnostic accuracy over CA19-9 alone for PDAC *vs* benign disease (increase in AUC 0.03 [95% CI −0.01 to 0.07]; τ^2^ 0.00 [0.00–0.01]; I^2^ 60% [0–94%]; Supplementary Fig. S5B). However, for PDAC *vs* healthy controls, a significant increase in the AUC was observed when combining THBS2 with CA19-9 in a prediction model (increase in AUC 0.07 [95% CI 0.03–0.11]; τ^2^ 0.00 [0.00–0.01]; I^2^ 32% [0–95%]; Supplementary Fig. S5C). For both comparisons, confidence intervals for I^2^ were very wide, indicating high uncertainty around the proportion of total variability that is due to between-study heterogeneity. However, the τ^2^ statistic and its corresponding confidence interval indicated low between-study heterogeneity.

### TIMP-1

Like THBS2, TIMP-1 was significantly less accurate than CA19-9 for PDAC *vs* healthy controls (rDOR 0.33 [95% CI 0.19–0.58]; p < 0.0001), and for PDAC *vs* benign disease (rDOR 0.12 [95% CI 0.06–0.26]; p < 0.0001). TIMP-1 performed markedly worse for PDAC *vs* benign disease compared with PDAC *vs* healthy controls (AUSROC 0.73 *vs* 0.92; rDOR 0.13 [95% CI 0.06–0.30]; p < 0.0001). Results were similar when restricting the analysis to comparative studies only (Supplementary Figs. S6 and S7).

### OPN

Five studies provided AUC values for the detection of PDAC by OPN with healthy controls as the control population (pooled AUC 0.82 [95% CI 0.67–0.91]). Substantial between-study heterogeneity was observed in this meta-analysis (τ^2^ 0.35 [95% CI 0.09–3.09]), although the confidence interval for τ^2^ was wide and was compatible with both modest and very high between-study heterogeneity. For PDAC *vs* healthy controls, there was strong evidence that OPN had a lower accuracy compared with CA19-9 (AUSROC 0.87 *vs* 0.93; rDOR 0.33 [95% CI 0.17–0.64]; p < 0.0001; Fig. 4A). Similarly, CA19-9 outperformed OPN for PDAC *vs* benign disease (AUSROC 0.89 *vs* 0.74; rDOR 0.24 [95% CI 0.06–0.89]; p < 0.0001). With PDAC *vs* healthy controls as the reference group, the performance of OPN was significantly lower for PDAC *vs* benign disease (AUSROC 0.89 *vs* 0.71; rDOR 0.17 [95% CI 0.08–0.38]; p < 0.0001).

### ICAM-1

The discriminatory power of ICAM-1 was similar to CA19-9 when assessing their performance in PDAC *vs* healthy controls (AUSROC 0.83 *vs* 0.85), and there was no evidence that ICAM-1 had a higher accuracy compared to CA19-9 (p = 0.53). However, there was evidence of a lower accuracy (p = 0.003) of ICAM-1 for PDAC *vs* benign disease compared with PDAC *vs* healthy controls, and ICAM-1 demonstrated both a significantly lower test positivity rate (p < 0.0001) and a lower overall accuracy than CA19-9 (AUSROC 0.68 *vs* 0.91; rDOR 0.11 [95% CI 0.01–1.15]; p < 0.0001) when assessed as a diagnostic biomarker for PDAC *vs* benign disease (Fig. 3B).

### IGFBP2

IGFBP2 had the lowest accuracy of all included proteins in distinguishing PDAC from healthy controls with a pooled AUC of 0.75 (95% CI 0.58–0.86). Although moderate between-study heterogeneity was observed in this meta-analysis (τ^2^ 0.18 [95% CI 0.03–2.82]), no definite inference about the extent of heterogeneity can be drawn due to a wide confidence interval. In HSROC meta-regression analyses, IGFBP2 performed markedly worse than CA19-9, both as a diagnostic biomarker for PDAC *vs* healthy controls (AUSROC 0.72 *vs* 0.90; rDOR 0.16 [95% CI 0.04–0.55]; p < 0.0001), and as a diagnostic biomarker for PDAC *vs* benign disease (0.68 *vs* 0.86; rDOR 0.22 [95% CrI 0.04–1.28]; p < 0.0001).

### Clinical implications

The clinical implications of implementing blood-based protein biomarkers as a triage test in the management of suspected PDAC are summarized in Supplementary Table S8. In a hypothetical cohort of 1000 patients with suspected PDAC, assuming a prevalence of 30%, the use of MIC-1 — the best performing protein — as a triage test would correctly classify 186 out of 300 patients with PDAC (ie, 114 PDAC patients would be missed), whereas 154 out of 700 patients without PDAC would be incorrectly classified as having PDAC. The results for other pre-test probabilities are provided in Supplementary Table S8.

### Added clinical value of protein biomarkers to CA19-9

**Fig. 5:**
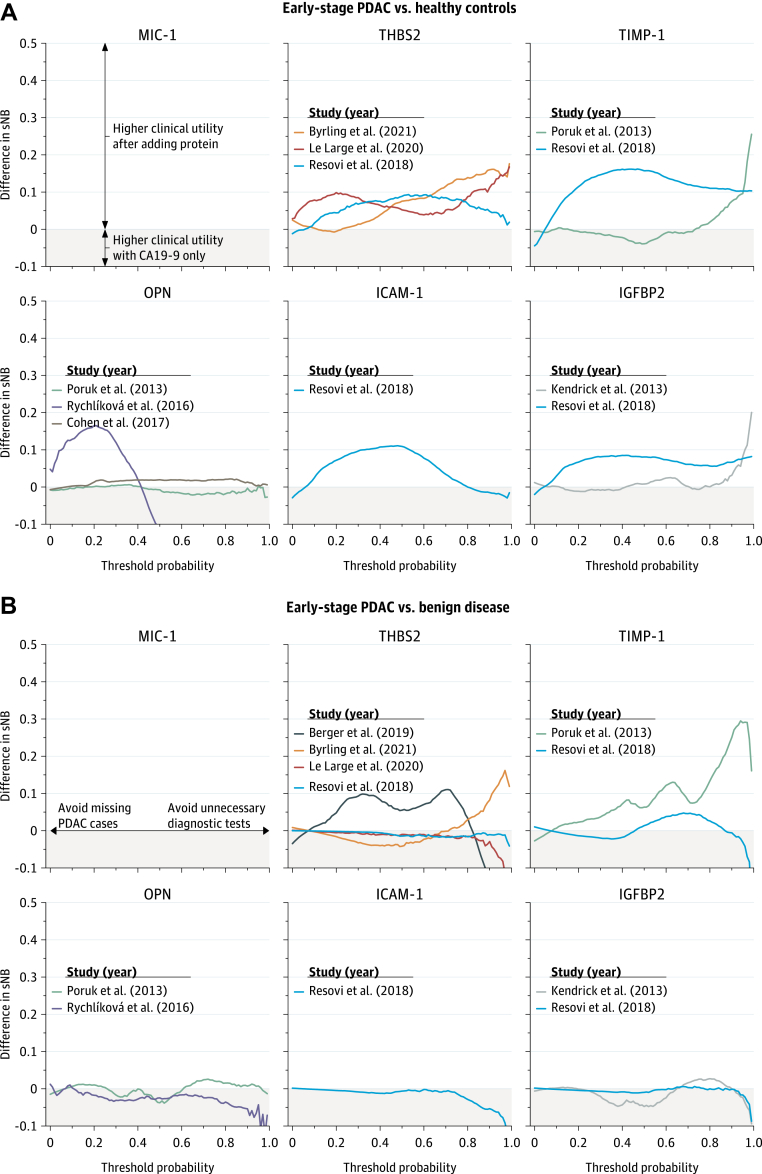
**Incremental clinical utility of protein biomarkers in individual studies.** PDAC indicates pancreatic ductal adenocarcinoma; sNB, standardized net benefit. The sNB can have negative values (clinical harm), and typically ranges between 0 (no clinical utility) and 1 (maximum clinical utility). The threshold probability of disease indicates the minimum risk of disease a patient needs to have before certain interventions are considered. For instance, if a clinician performs 100 biopsies to find 20 PDAC cases, then the threshold probability is 20%, and the benefit of detecting one PDAC case is deemed to be ([100-20]/20=) 4 times higher than the harm of performing one unnecessary biopsy. A difference in sNB of 0 indicates that adding a protein biomarker to CA19-9 in a prediction model has no added clinical value over using CA19-9 alone. A difference in sNB of lower than 0 (grey shaded region in the plots) indicates that adding a protein biomarker to CA19-9 would result in clinical harm compared with using CA19-9 only in the management of suspected PDAC.

In a re-analysis of eight primary studies that provided patient-level data,^24^^,^^25^^,^^28^^,^^33^^,^^38^^,^^43^^,^^48^^,^^49^ the addition of protein biomarkers to CA19-9 resulted in either minor improvements or limited decreases in clinical utility, both for early-stage PDAC *vs* healthy controls (Fig. 5A) and for early-stage PDAC *vs* benign disease (Fig. 5B). Differences in clinical utility were negligible at relevant decision thresholds (5–30%). Results were consistent across several sensitivity analyses, in which different pre-test probabilities were assumed (Supplementary Fig. S8A–D). The discrimination, calibration, and clinical utility of CA19-9, protein biomarkers, and their combination for all studies that provided patient-level data are provided in Supplementary Figs. S9–S16 for all-stage PDAC *vs* benign disease.

## Discussion

This first meta-analysis on the accuracy of diagnostic blood-based proteins in comparison to CA19-9 in patients with PDAC, identified the following potentially promising proteins: MIC-1, THBS2, TIMP-1, OPN, ICAM-1, and IGFBP2. These proteins did not outperform CA19-9 in differentiating between PDAC and benign disease, and resulted in limited added clinical value when combined with CA19-9 in a prediction model. The identification and implementation of accurate diagnostic biomarkers is crucial for fast and efficient diagnosis, to ultimately improve outcomes and prevent unnecessary interventions for patients with suspected PDAC.

Studies included in this systematic review and meta-analysis which analyzed protein expression in pre-treatment blood samples of patients with PDAC *vs* non-PDAC controls, generally found divergent results, and many of the investigated protein biomarkers have not been evaluated across multiple studies. Nevertheless, research suggests that these proteins play a significant role in PDAC carcinogenesis related activities, such as proliferation, migration, apoptosis, or angiogenesis, and therefore could prove to be of diagnostic value for patients with PDAC.^50^ For instance, MIC-1, a macrophage inhibitory cytokine, is dysregulated under pathological conditions such as inflammation and in the presence of various cancers and it could indeed be an indicator for PDAC development.^45^ TIMP-1 plays a crucial role in extracellular matrix composition, and aberrant expression of this protein could indeed indicate stroma development.^52^ These proteins could possibly be further evaluated for their predictive or prognostic capability, but, considering their pooled diagnostic accuracy compared to CA19-9, are not likely to singularly replace CA19-9 as diagnostic biomarker in the near future.

MIC-1 was slightly more accurate than CA19-9 in the pooled comparison between patients with PDAC and healthy controls, a group which does not accurately reflect the clinical setting in which these biomarkers most likely would be implemented. In further analyses of MIC-1, THBS2, TIMP-1, OPN, ICAM-1, and IGFBP2 in cohorts with PDAC patients *vs* benign disease, the performance of all biomarkers deteriorates significantly, and CA19-9 consistently showed a superior diagnostic performance. This could be because these proteins are aberrantly expressed in both inflammatory and malignant processes. As the majority of the investigated non-PDAC control groups consists of healthy controls, the protein biomarkers are often not validated in clinically representative cohorts and therefore their accuracy may be overestimated due to spectrum effects.^53^^,^^54^ To accurately map a clinical setting, a cohort consisting of patients with PDAC and patients with benign pancreatic diseases is indispensable, as distinguishing malignant from benign disease forms an important aspect of the clinical diagnostic problem. Few studies indeed include this rationale and focus primarily on PDAC *vs* healthy controls, in which the protein biomarkers generally will perform better than CA19-9, as serum levels of this protein biomarker will not be elevated in both groups, contrary to a comparison of malignant and benign conditions.^55^

Considering the methodological deficiencies described above, it is of value to propose recommendations for future diagnostic biomarker research for PDAC patients.

First, the design of the study should match the clinical setting in which the test is intended to be used. As such, discovery and particularly validation of novel serum or plasma protein biomarkers for PDAC should primarily be conducted using benign pancreatic diseases as control group, rather than solely healthy controls. In general, use of a single-gate design is recommended, and studies should aim to assess biomarkers in patients with suspected PDAC as their target population. Specifically, this means recruiting a cohort of patients with suspected PDAC (ie, prior to final diagnosis), rather than using a multiple-gate design (‘case–control design’) in which patients with PDAC and controls (eg, chronic pancreatitis) are recruited separately.

Second, studies that aim to discover or validate novel protein biomarkers should preferably also assess the diagnostic performance of previously discovered promising biomarkers. This approach would facilitate more accurate estimation of the diagnostic performance of protein biomarkers across a range of studies.

Third, novel biomarkers should be assessed for their added value to CA19-9 and other routinely measured biomarkers. High diagnostic accuracy of a single protein biomarker is, on its own, generally insufficient to warrant further consideration of the protein in external validation or clinical impact studies. Rather, novel protein biomarkers should demonstrate sufficient added value – in terms of diagnostic accuracy and potential clinical utility – when added to prediction models using inexpensive and routinely measured markers, such as clinical symptoms, CA19-9, and bilirubin.

Fourth, clinical prediction models should adhere to minimum sample size requirements, both during development^56^ and external validation,^57^ to prevent overoptimism in model performance estimates, increase the likelihood of adequate performance at external validation, and allow accurate estimation of key performance metrics of the model. In general, prediction models should be developed, validated, and reported in accordance with the TRIPOD-statement.^58^

Lastly, the cost-effectiveness and impact of successfully externally validated prediction models on clinical decision making and patient outcomes should be evaluated in prospective studies.

The evidence base of this meta-analysis should be assessed in light of several limitations. First, there is considerable heterogeneity among the included studies. The most important source of heterogeneity was whether the control group consisted of healthy controls or benign diseases. This factor alone explained 66% (median R^2^ for all biomarkers; range, 24–82%) of the heterogeneity that was observed in the AUC of the biomarkers. To account for this source of heterogeneity and explore its impact on the diagnostic accuracy of biomarkers, we performed stratified analyses and meta-regression analyses, respectively. However, due to a lack of studies we could not account for the specific composition of the benign disease control group. Thus, it is not possible to conclude whether the performance of biomarkers varies per patient group; eg, to specify whether MIC-1 accurately distinguishes PDAC from chronic pancreatitis but cannot accurately distinguish PDAC from acute pancreatitis or a benign biliary obstruction. For this reason, our meta-analysis merges the benign group as one group, even though important clinically distinct benign diseases then remain unacknowledged. The impact of other potential sources of heterogeneity, such as type of blood sample (serum *vs* plasma [R^2^, 0.63%]) and type of test comparison (direct *vs* indirect comparisons), was limited and did not change the conclusions of our analyses.

Second, the use of optimal threshold selection methods (eg, with the Youden index) for protein biomarkers could have resulted in overoptimistic performance estimates of the proteins compared with CA19-9 in the HSROC analysis. Therefore, a separate meta-analysis of the AUCs was also conducted, as this approach is invariant to data-driven cut-off selection methods.

In conclusion, the present findings suggest that not one protein biomarker outperforms CA19-9 as a diagnostic marker for distinguishing PDAC from benign disease in clinically relevant studies, and that these single protein biomarkers offer limited added clinical value over CA19-9 alone. There is room for improvement of the pipeline for biomarker discovery. Initially, biomarkers should be critically assessed for their added value to readily available clinical information and biomarkers, especially CA19-9. Subsequently, the clinical consequences of implementing a novel test should prospectively be assessed to determine the clinical utility of the test in addition to or relative to routinely measured markers and the current diagnostic pathway. These outcomes should intensively be researched and reported, to benefit diagnostics and ultimately treatment and prognosis for patients suffering from PDAC.

## Contributors

The study was designed by L.N.C.B. and M.A. L.N.C.B. and M.A. collected and verified the data. M.A. performed the statistical analyses. All authors were involved in interpretation of the data, and contributed to drafting the manuscript and critical revision of the manuscript with regard to important intellectual content. All authors approved the final version of the manuscript prior to submission.

## Data sharing statement

Individual patient data are available upon permission from corresponding authors of the primary studies. Aggregate data are provided in the main text and the supplemental material.

## Declaration of interests

We declare no competing interests.

## Appendix A Supplementary data

Supplementary Figures S1–S18, Tables S1–S8, and Appendices 1–3

Supplementary Figures S1–S18, Tables S1–S8, and Appendices 1–3 Captions
