# Supplementary material for: Diagnostic accuracy and added value of blood-based protein biomarkers for pancreatic cancer: A meta-analysis of aggregate and individual participant data
Source: eClinicalMedicine. 2022 Nov 24;55:101747. doi: 10.1016/j.eclinm.2022.101747 (PMC9706531; doi:10.1016/j.eclinm.2022.101747)
Supplement: Supplementary Figures S1–S18, Tables S1–S8, and Appendices 1–3 [file mmc1.docx]

**Supplementary methods**

Diagnostic two-by-two tables were extracted from the primary studies or reconstructed using either reported summary estimates or by contacting the authors of the primary studies to obtain the original study data. For each study, the index tests, population characteristics, test positivity thresholds, and the number of true-positives, false-positive, false-negatives, and true-negatives were extracted. The diagnostic two-by-two tables were used to estimate the sensitivity, specificity, and corresponding 95% confidence intervals using either modified Wilson or Agresti-Coull intervals, depending on the number of cases and controls.^1^

We assessed four aspects of the performance of protein biomarkers:

1. The diagnostic odds ratio of the biomarkers and the shape of the summary receiver operating characteristic (ROC) curve, if studies reported different cutoffs.
2. The pooled sensitivity and specificity of the biomarkers, if four or more studies reported sensitivity and specificity estimates at a common cutoff.
3. The overall diagnostic performance of the biomarkers (i.e., the area under the ROC curve [AUC]).
4. The potential clinical utility of the biomarkers.

*HSROC model*

The diagnostic performance of each biomarker was analyzed with the Rutter and Gatsonis hierarchical summary receiver operating characteristic (HSROC) model to produce summary receiver operating characteristic (SROC) curves, and to calculate the diagnostic odds ratio as a summary measure of overall accuracy. Frequentist HSROC models were only fitted if data from four or more studies were available, or if at least three studies were available per covariate level in HSROC meta-regression models. If a paucity of studies precluded convergence of the complete HSROC model despite optimization of starting values for the parameter estimates, or if data from less than 10 studies were available, a reduced HSROC model was fitted by removing the shape parameter in the model to produce a symmetrical SROC curve.^2^ Potential sources of heterogeneity were explored with meta-regression by extending the symmetric HSROC model with study-level covariates to assess their effect on the threshold and accuracy parameters of the SROC curve, while assuming the shape parameter and the variance of the random effects for the accuracy and threshold parameters to be common for each covariate level to facilitate convergence of the model.^2^ The likelihood ratio χ^2^ of competing models was used to guide selection of the final model and to assess the inclusion and exclusion of covariates for their statistical significance. All *P* values reported for HSROC meta-regression analyses are derived from likelihood ratio tests rather than Wald statistics.^3^

Bayesian symmetric HSROC models were fitted using Markov Chain Monte Carlo simulation and the rjags package in R, if the variance of the accuracy parameter was estimated with high uncertainty in a frequentist HSROC model (i.e., using PROC NLMIXED in SAS [version 9.4]), and the gradients for all other model parameters were close to zero. All (hyper-)prior distributions and other aspects of the rjags program were taken from the supplementary material for Chapter 11 of the Cochrane Handbook for Systematic Reviews of Diagnostic Test Accuracy (specifically, model A6.2 and model A6.4). However, in our rjags program, we removed the shape parameter and the covariate for the shape parameter (respectively, the beta and delta parameter in the HSROC meta-regression model), as these parameters cannot reliably be estimated when data from fewer than ten studies are available. In addition, because we compared two index tests with each other, while model A6.4 compares three index tests with each other, we removed covariates for the third test from our model.

Frequentist likelihood ratio tests are presented for all HSROC meta-regression models if the convergence criterion was satisfied. If the variance of the accuracy parameter was estimated with high uncertainty with PROC NLMIXED in SAS, then a Bayesian HSROC meta-regression analysis was performed for estimation of model parameters, while a frequentist likelihood ratio test with PROC NLMIXED was used for statistical testing. For this likelihood ratio test, the difference in the -2 log likelihood of the 'full model’ (with the covariate) and the -2 log likelihood of the ‘reduced model’ (without the covariate) was used along with the appropriate number of degrees of freedom (ie, the difference in the number of model parameters) to obtain a *P* value.

The area under the SROC curve (AUSROC) was estimated using numerical integration. Confidence and credible intervals of the AUSROC could readily be estimated using the corresponding confidence and credible intervals of the accuracy parameter, as we assumed a symmetric shape of the SROC curve. Thus, the lower limit of the 95% CI of the AUSROC is the area under a symmetric SROC curve that is defined by the lower limit of the 95% CI of the accuracy parameter. The same applies, *mutatis mutandis*, for the upper limit of the 95% CI of the AUSROC.

*Bivariate model*

For THBS2 *vs* CA19-9, we additionally compared their pooled sensitivity and specificity at the commonly used cut-offs of 42 ng/mL and 37 U/mL, respectively. For this purpose, we initially used the generalized linear mixed modeling approach of Chu and Cole.^4^ This approach gives less biased results than the standard bivariate model of Reitsma et al, which uses an inappropriate normal approximation.^5^ Starting parameters for the model were taken from initial attempts at fitting the model using the metandi module in Stata (version 17.0). However, as these models failed to converge in both Stata and SAS, and provided unreliable estimates of the between-study correlation, we performed a Bayesian bivariate meta-regression analysis instead.^6^ For this model, we used the same rjags program as in appendix 6.3 of the supplementary material for Chapter 11 of the Cochrane Handbook for Systematic Reviews of Diagnostic Test Accuracy.

*Random-effects meta-analysis of the AUC*

For both the aggregate data meta-analysis of area under the curve (AUC) estimates and the two-stage individual participant data meta-analysis, we performed a random-effects meta-analysis of the logit-transformed AUC estimates with a restricted maximum likelihood estimator and Jackson’s modification of the Hartung-Knapp-Sidik-Jonkman variance correction. If the confidence interval of the AUC was not reported, this confidence interval was approximated using Newcombe’s method (implemented in the metamisc package in R).^7,8^

A logit transformation (rather than an arcsine transformation or no transformation at all) was used as this is the best approach to ensure a normal distribution of the AUC.^9^ We used a restricted maximum likelihood estimator to estimate the between-study variance, which outperforms other estimation methods, including the standard DerSimonian-Laird method, for meta-analyses of continuous outcomes.^10,11^ We used Jackson’s modification of the Hartung-Knapp/Sidik-Jonkman variance correction to improve control of the Type I error rate and achieve coverage closer to the nominal 95%.^12-14^ The same approach (REML estimator with Jackson’s modification of the Hartung-Knapp/Sidik-Jonkman variance correction) is also the recommended approach for two-stage random-effects IPD meta-analyses.^15^ Prediction intervals were not calculated, as their performance was expected to be poor due to high heterogeneity and a lack of studies.^16,17^ All aggregate and two-stage IPD random-effects meta-analyses of the AUC were performed using the metafor package (version 3.8-1) in R.

*Potential clinical impact of biomarkers*

To assess the potential clinical impact of using blood-based protein biomarkers, the median observed specificity of the biomarker was used to derive a corresponding sensitivity estimate from the HSROC model output.

We expressed the clinical implications of the biomarkers in the following way:

1. Test positive:
   1. False-positives: “No PDAC but given diagnostic intervention”
   2. True-positives: “PDAC and diagnostic intervention performed”
   3. True-positives/(true-positives + false-positives): “Proportion of diagnostic interventions performed correctly”
2. Test negative:
   1. True-negatives + false-negatives: “Diagnostic intervention not performed”
   2. False-negatives: “PDAC cases missed”
   3. True-negatives/[true-negatives + false-negatives]): “Proportion of diagnostic interventions avoided correctly”

*Added value of protein biomarkers*

The added diagnostic value of adding a protein biomarker was assessed by calculating the difference in AUC between (1) a prediction model using both CA19-9 and another protein biomarker, and (2) a prediction model using CA19-9 only. As these two AUC estimates were expected to be correlated, the 95% CI of this difference was estimated using either bootstrapping, if IPD data were available, or using the Hanley-McNeil method (see the formula below), if only aggregate data were available.^18^

$$z=\frac{{AUC}_{1} - {AUC}_{2}}{\sqrt{{({SE}_{1})}^{2}+ {({SE}_{2})}^{2} -2 \cdot r\cdot{SE}_{1} \cdot{SE}_{2}}}$$

In this formula, the critical ratio *z* is calculated using (1) the AUC of the prediction model using CA19-9 and another protein biomarker (AUC_1_); (2) its corresponding standard error (SE_1_), as reported by the original authors of the primary study or approximated using Newcombe’s method; (3) the AUC of CA19-9 (AUC_2_); (4) its corresponding standard error (SE_2_); (5) the correlation (*r*) between AUC_1_ and AUC_2_. This correlation parameter was obtained by calculating the median observed correlation in studies that provided IPD data.

The study data shared by corresponding authors was used to estimate the added clinical value of proteins over CA19-9 alone, and to identify non-linear relationships that should be considered when building clinical prediction models using CA19-9 and blood-based proteins. Non-linear relationships between continuous variables (i.e., the concentration of CA19-9 and blood-based proteins) and the log odds of having PDAC were explored using restricted cubic splines with three knots at the marker’s 10^th^, 50^th^, and 90^th^ percentile. A formal linearity test was also performed using the anova.rms function in R (version 4.2.0).

The incremental clinical utility of proteins was estimated through overoptimism-corrected decision curve analyses. Briefly, the overoptimism, as estimated from 1000 bootstrap replicates, was subtracted from the LOESS-smoothed standardized net benefit (sNB) at each decision threshold. Specifically, the following steps were undertaken:

1. A decision curve analysis was performed for a clinical prediction model that was developed and tested in the original study dataset using (1) CA19-9 only, and (2) CA19-9 and a blood-based protein. The standardized net benefit was obtained at each decision threshold (ranging from 0% to 100% with increments of 1%) and smoothed using nonparametric LOESS regression to prevent statistical artifacts and produce monotonically non-increasing curves. The resulting estimates were defined as the apparent performance of the models.
2. In total, 1000 bootstrap samples were subsequently drawn with replacement from the original study dataset. For each bootstrap sample, two logistic regression models were fitted: one using only CA19-9, and one using CA19-9 and a protein. These models were evaluated in the same bootstrap sample (the ‘training sample’) and in the original study dataset (the ‘testing sample’). At each decision threshold, the sNB was compared between the training and the testing sample, and the difference in sNB was defined as the overoptimism in the model’s performance.
3. At each decision threshold, the average of the overoptimism estimates was calculated and subtracted from the apparent performance to obtain overoptimism-corrected sNB estimates at that threshold.
4. The difference between the overoptimism-corrected sNB estimates for CA19-9 and the combination of CA19-9 and a blood-based protein was calculated to obtain the incremental clinical utility of protein biomarkers.

*Assessing heterogeneity and proportion of variance explained*

Between-study heterogeneity in the AUC was assessed using τ^2^ (reported on the logit scale), whereas the proportion of variability due to between-study heterogeneity was assessed using the I^2^ statistic. Confidence intervals for these two measures were derived using the confint function in the metafor package.

We used meta-regression analyses to identify possible sources of heterogeneity, and used an R^2^ statistic to study the potential importance of predictor variables (e.g., serum *vs* blood), as described previously.^19^ This R^2^ statistic is defined as follows:

$$R^{2}=\frac{\tau_{explained}^{2}}{\tau_{total}^{2}}$$

In this formula, τ^2^_explained_ is the variance that is explained by taking into account a certain predictor variable, while τ^2^_total_ is the variance in a 'standard' meta-analysis that does not take predictor variables into account.

HSROC meta-regression analyses were also performed to study the impact of limiting the analysis to comparative studies only.

**References**

1. Brown LD, Cai TT, DasGupta A. Interval estimation for a binomial proportion. *Statistical science* 2001; **16**(2): 101-33.

2. Takwoingi Y, Guo B, Riley RD, Deeks JJ. Performance of methods for meta-analysis of diagnostic test accuracy with few studies or sparse data. *Statistical methods in medical research* 2017; **26**(4): 1896-911.

3. Agresti A. Categorical data analysis: John Wiley & Sons; 2003.

4. Chu H, Cole SR. Bivariate meta-analysis of sensitivity and specificity with sparse data: a generalized linear mixed model approach. *Journal of clinical epidemiology* 2006; **59**(12): 1331.

5. Reitsma JB, Glas AS, Rutjes AW, Scholten RJ, Bossuyt PM, Zwinderman AH. Bivariate analysis of sensitivity and specificity produces informative summary measures in diagnostic reviews. *Journal of clinical epidemiology* 2005; **58**(10): 982-90.

6. Riley RD, Abrams KR, Sutton AJ, Lambert PC, Thompson JR. Bivariate random-effects meta-analysis and the estimation of between-study correlation. *BMC Medical Research Methodology* 2007; **7**(1): 1-15.

7. Debray TP, Damen JA, Riley RD, et al. A framework for meta-analysis of prediction model studies with binary and time-to-event outcomes. *Statistical methods in medical research* 2019; **28**(9): 2768-86.

8. Newcombe RG. Confidence intervals for an effect size measure based on the Mann–Whitney statistic. Part 2: asymptotic methods and evaluation. *Statistics in medicine* 2006; **25**(4): 559-73.

9. Snell KI, Ensor J, Debray TP, Moons KG, Riley RD. Meta-analysis of prediction model performance across multiple studies: Which scale helps ensure between-study normality for the C-statistic and calibration measures? *Statistical Methods in Medical Research* 2018; **27**(11): 3505-22.

10. Langan D, Higgins JP, Jackson D, et al. A comparison of heterogeneity variance estimators in simulated random‐effects meta‐analyses. *Research synthesis methods* 2019; **10**(1): 83-98.

11. Veroniki AA, Jackson D, Viechtbauer W, et al. Methods to estimate the between‐study variance and its uncertainty in meta‐analysis. *Research synthesis methods* 2016; **7**(1): 55-79.

12. Jackson D, Law M, Rücker G, Schwarzer G. The Hartung‐Knapp modification for random‐effects meta‐analysis: a useful refinement but are there any residual concerns? *Statistics in medicine* 2017; **36**(25): 3923-34.

13. Knapp G, Hartung J. Improved tests for a random effects meta‐regression with a single covariate. *Statistics in medicine* 2003; **22**(17): 2693-710.

14. Röver C, Knapp G, Friede T. Hartung-Knapp-Sidik-Jonkman approach and its modification for random-effects meta-analysis with few studies. *BMC medical research methodology* 2015; **15**(1): 1-7.

15. Riley RD, Debray TP, Morris TP, Jackson D. The Two‐stage Approach to IPD Meta‐Analysis. *Individual Participant Data Meta‐Analysis: A Handbook for Healthcare Research* 2021: 87-125.

16. Partlett C, Riley RD. Random effects meta‐analysis: coverage performance of 95% confidence and prediction intervals following REML estimation. *Statistics in medicine* 2017; **36**(2): 301-17.

17. Egger M, Higgins JP, Smith GD. Systematic Reviews in Health Research: Meta-Analysis in Context: John Wiley & Sons; 2022.

18. Hanley JA, McNeil BJ. A method of comparing the areas under receiver operating characteristic curves derived from the same cases. *Radiology* 1983; **148**(3): 839-43.

19. Higgins JP, Li T. Exploring Heterogeneity. *Systematic Reviews in Health Research: Meta‐Analysis in Context* 2022: 185-203.

**Supplementary results**

**Supplementary figure 1. Quality assessment of studies included in the diagnostic meta-analysis.**

PS, patient selection; IT, index test; RS, reference standard; FT, flow and timing.

**Supplementary figure 2. Pooled AUC of protein biomarkers *vs* CA19-9 in direct, head-to-head comparisons.**

Meta-analyses were not performed if the AUC of only 2 studies were available, or if substantial between-study heterogeneity was present in a meta-analysis of 3 studies. A pooled AUC of CA19-9 across all included studies is presented for comparative purposes. PDAC indicates the number of included PDAC patients; BD, number of included patients with benign disease; REML, restricted maximum likelihood; mHKSJ, Jackson’s modification of the Hartung-Knapp/Sidik-Jonkman variance correction.

**Supplementary figure 3. AUC of blood-based proteins and CA19-9 as diagnostic biomarkers for PDAC *vs* healthy controls.**

**Supplementary figure 3. (Continued).**

**R**EML, restricted maximum likelihood; mHKSJ, Jackson’s modification of the Hartung-Knapp/Sidik-Jonkman variance correction. τ^2^ is reported on the logit scale; the subscript under Cochran’s Q statistic indicates the degrees of freedom, i.e., the number of studies minus 1.

**Supplementary figure 4. AUC of blood-based proteins and CA19-9 as diagnostic biomarkers for PDAC *vs* benign disease.**

**Supplementary figure 4. (Continued).**

REML, restricted maximum likelihood; mHKSJ, Jackson’s modification of the Hartung-Knapp/Sidik-Jonkman variance correction. τ^2^ is reported on the logit scale; the subscript under Cochran’s Q statistic indicates the degrees of freedom, i.e., the number of studies minus 1.

**Supplementary figure 5. Incremental diagnostic accuracy (∆AUC) from adding blood-based proteins to CA19-9.**

Increase in AUC when adding a protein biomarker to a prediction model using CA19-9 alone. For instance, if CA19-9 has an AUC of 0.80, and a prediction model with both CA19-9 and THBS2 has an AUC of 0.83, then the increase in AUC (∆AUC) is 0.83 – 0.80 = 0.03. Meta-analyses were only performed if estimates from three or more studies were available. Confidence intervals for the ∆AUC were estimated using bootstrap resampling or the Hanley-McNeil method, as the AUC of the two models were expected to be correlated.

**Supplementary figure 6. Relative diagnostic accuracy of proteins and CA19-9, restricted to comparative studies.**

Individual studies are presented as symbols, and head-to-head comparisons within studies are indicated with grey lines connecting two symbols. An rDOR lower than 1 indicates that the protein biomarker has a lower diagnostic accuracy than CA19-9. CrI, credible intervals (derived from Bayesian hierarchical models).

**Supplementary figure 7. Relative diagnostic accuracy of protein biomarkers for PDAC *vs* healthy controls and PDAC *vs* benign disease, restricted to comparative studies**.

An rDOR lower than 1 indicates that the protein biomarker has a lower accuracy for PDAC *vs* benign disease compared with the protein’s accuracy for PDAC *vs* healthy controls. CrI, credible interval (derived from Bayesian hierarchical models).

**Supplementary figure 8. Incremental clinical value of blood-based proteins compared with CA19-9 for PDAC *vs* benign disease, assuming different pre-test probabilities for PDAC**.

**Supplementary figure 9. Discrimination, calibration, and clinical utility of THBS2 in Le Large et al. (2020) for PDAC *vs* benign disease.**

Overoptimism-corrected calibration curves and decision curves were obtained through 1000 repeats of Harrell’s bootstrap resampling procedure. In figure B, the gray oblique line indicates perfect calibration (intercept of 0 and a slope of 1).

**Supplementary figure 10. Discrimination, calibration, and clinical utility of OPN in Rychlíková et al. (2016) for PDAC *vs* benign disease.**

Overoptimism-corrected calibration curves and decision curves were obtained through 1000 repeats of Harrell’s bootstrap resampling procedure. In figure B, the gray oblique line indicates perfect calibration (intercept of 0 and a slope of 1).

**Supplementary figure 11. Discrimination, calibration, and clinical utility of OPN in Cohen et al. (2017) for PDAC *vs* healthy controls.**

Overoptimism-corrected calibration curves and decision curves were obtained through 1000 repeats of Harrell’s bootstrap resampling procedure. In figure B, the gray oblique line indicates perfect calibration (intercept of 0 and a slope of 1).

**Supplementary figure 12. Discrimination, calibration, and clinical utility of OPN and TIMP-1 in Poruk et al. (2013) for PDAC *vs* benign disease.**

Overoptimism-corrected calibration curves and decision curves were obtained through 1000 repeats of Harrell’s bootstrap resampling procedure. In figure B, the gray oblique line indicates perfect calibration (intercept of 0 and a slope of 1). Interc., intercept.

**Supplementary figure 13. Discrimination, calibration, and clinical utility of IGFBP2 in Kendrick et al. (2013) for PDAC *vs* benign disease.**

Overoptimism-corrected calibration curves and decision curves were obtained through 1000 repeats of Harrell’s bootstrap resampling procedure. In figure B, the gray oblique line indicates perfect calibration (intercept of 0 and a slope of 1).

**Supplementary figure 14. Discrimination, calibration, and clinical utility of THBS2 in Berger et al. (2019) for PDAC *vs* benign disease.**

**Supplementary figure 15. Discrimination, calibration, and clinical utility of THBS2 in Byrling et al. (2021) for PDAC *vs* benign disease.**

The intercept and slope for THBS2 were estimated using 5000 bootstrap replicates, as the performance of THBS2 was highly variable due to a low number of patients.

**Supplementary figure 16. Discrimination and calibration of THBS2, TIMP-1, ICAM-1, and IGFBP2 in Resovi et al. (2018) for PDAC *vs* benign disease.**

Prediction models using CA19-9 and other blood-based proteins are not shown, as these proteins did not add significant value to a prediction model consisting of only CA19-9 (4 *df* likelihood ratio test of CA19-9 *vs* [CA19-9 + THBS2 + TIMP-1 + ICAM-1 + IGFBP2]: *P*=0.17; increase in the bootstrap-corrected R^2^: 0.0001).

**Supplementary figure 17. Non-linear relationships between CA19-9 and PDAC in individual studies.**

Non-linear relationships between CA19-9 and PDAC were modelled using restricted cubic splines with three knots at the 10th, 50th, and 90th percentile of CA19-9. For the study of Rychlíková et al., CA19-9 was winsorized at the 99th quantile to facilitate convergence of the model. For the non-linearity Wald test, a *P*<0.05 indicates that there is evidence for a non-linear relationship between CA19-9 and the log odds of having PDAC. If CA19-9 is not modelled as a nonlinear term in a clinical prediction model (using, eg, restricted cubic splines or multivariable fractional polynomials), then the AUC, calibration, and clinical utility of the model will be lower than if these nonlinear relationships are taken into account, assuming that the sample size is sufficient to support these additional model parameters.

**Supplementary figure 18. Non-linear relationships between proteins and PDAC in individual studies.**

Non-linear relationships between proteins and PDAC were modelled using restricted cubic splines with three knots at their respective 10th, 50th, and 90th percentiles. For the non-linearity Wald test, a *P*<0.05 indicates that there is evidence for a non-linear relationship between CA19-9 and the log odds of having PDAC.

| **Supplementary table 1. Eligibility criteria.** |  |
| --- | --- |
| **Inclusion criteria** | **Exclusion criteria** |
| **Study type** |  |
| - Studies written in English  - Human studies  - Randomized controlled trial  - Non-randomized controlled trial  - Prospective cohort study  - Retrospective cohort study  - Case series with 10 or more PDAC patients and 10 or more controls  - Studies involving only a subset of relevant patients will be included if data is subdivided and contains ≥10 relevant patients | - Animal studies  - Systematic review  - Meta-analysis  - Case series with less than 10 patients  - Conference abstract |
| **Included participants** |  |
| - Histopathological diagnosis of pancreatic ductal adenocarcinoma  - Primary tumor  - Disease stages, or T and N classification  - Age ≥ 18 year  - Male and female patients  - Pre-treatment blood samples | - No PDAC patients included  - Secondary tumor / metastasis of any other primary tumor |
| **Type of biomarker** |  |
| - Diagnostic protein biomarker  - Discriminative between PDAC and benign disease and/or healthy controls | - Prognostic or predictive biomarkers  - No protein biomarker studied (e.g., only miRNAs or ctDNA) |
| **Primary outcome** |  |
| - Area under the curve and/or sensitivity and specificity, specified per biomarker |  |

| **Supplementary table 2. Characteristics of included studies.** | | | | | | | | | | | | | |
| --- | --- | --- | --- | --- | --- | --- | --- | --- | --- | --- | --- | --- | --- |
| **Study, year** | **Study design** | **Comparison** | **No. of PDAC/**  **no. of controls** | **Benign group composition** | **Specimen** | **Biomarker** | **AUC (95% CI)** | **Threshold** | **TP** | **FP** | **FN** | **TN** |  |
| Berger et al, 2019 | Multiple-gate | PDAC *vs* healthy controls | 30/24 |  | Plasma | THBS2 | 0.73 (0.63-0.83) | 42 ng/mL | 15 | 1 | 15 | 23 |  |
|  |  |  |  |  |  | CA19-9 | 0.80 (0.80-0.89) | 55 U/mL | 19 | 1 | 11 | 23 |  |
| Byrling et al, 2021 | Single-gate | PDAC *vs* benign disease | 52/27 | Cystadenoma, CP, IPMN, AIP | Serum | THBS2 | - | 42 ng/mL | 31 | 12 | 21 | 15 |  |
|  |  |  |  |  |  | CA19-9 | - | 35 U/mL | 35 | 7 | 17 | 20 |  |
|  |  | PDAC *vs* healthy controls | 52/52 |  | Serum | THBS2 | 0.81 (0.71-0.90) | 42 ng/mL | 31 | 4 | 21 | 48 |  |
|  |  |  |  |  |  | CA19-9 | 0.83 (0.76-0.89) | 35 U/mL | 35 | 1 | 17 | 51 |  |
| Capello et al, 2016 | Multiple-gate | PDAC *vs* benign disease | 73/74 | CP, pancreatic cysts | Plasma | TIMP-1 | 0.74 (0.66-0.83) | - | 69 | 50 | 4 | 24 |  |
|  |  |  |  |  |  | IGFBP2 | 0.63 (0.54-0.72) | - | 16 | 4 | 57 | 70 |  |
|  |  |  |  |  |  | CA19-9 | 0.83 (0.75-0.91) | - | 69 | 55 | 4 | 19 |  |
|  |  | PDAC *vs* healthy controls | 73/60 |  | Plasma | TIMP-1 | 0.88 (0.81-0.96) | - | 30 | 3 | 43 | 57 |  |
|  |  |  |  |  |  | IGFBP2 | 0.80 (0.72-0.89) | - | 31 | 3 | 42 | 57 |  |
|  |  |  |  |  |  | CA19-9 | 0.88 (0.81-0.96) | - | 53 | 3 | 20 | 57 |  |
| Chang et al, 2009 | Multiple-gate | PDAC *vs* healthy controls | 52/43 |  | Plasma | OPN | - | - | 26 | 1 | 26 | 42 |  |
|  |  |  |  |  |  | CA19 | - | - | 13 | 1 | 39 | 42 |  |
| Cohen et al, 2017 | Multiple-gate | PDAC *vs* healthy controls | 221/182 |  | Plasma | OPN | 0.68 (0.63-0.74) | 334 ng/mL | 104 | 20 | 117 | 162 |  |
|  |  |  |  |  |  | CA19 | 0.89 (0.86-0.92) | 37 U/mL | 158 | 11 | 63 | 171 |  |
| Faca et al,  2008 | Multiple-gate | PDAC *vs* benign disease | 30/10 | CP | Serum | TIMP-1 | 0.88 (0.75-1.00) | 166 ng/mL | 26 | 1 | 4 | 9 |  |
|  |  |  |  |  |  | ICAM-1 | 0.92 (0.82-1.00) | 99 ng/mL | 29 | 5 | 1 | 5 |  |
|  |  |  |  |  |  | CA19 | 0.79 (0.63-0.95) | 51 U/mL | 24 | 3 | 6 | 7 |  |
|  |  | PDAC *vs* healthy controls | 30/20 |  | Serum | TIMP-1 | 0.89 (0.79-0.99) | 166 ng/mL | 26 | 5 | 4 | 15 |  |
|  |  |  |  |  |  | ICAM-1 | 0.75 (0.61-0.89) | 302 ng/mL | 20 | 7 | 10 | 13 |  |
|  |  |  |  |  |  | CA19 | 0.98 (0.94-1.00) | 24 U/mL | 28 | 2 | 2 | 18 |  |
| Hogendorf et al, 2018 | Single-gate | PDAC *vs* benign disease | 42/21 | CP | Serum | MIC-1 | 0.83 (0.72-0.93) | 2700 pg/mL | 31 | 5 | 11 | 16 |  |
|  |  |  |  |  |  | CA19 | 0.77 (0.65-0.88) | 36 U/mL | 24 | 2 | 18 | 19 |  |
| Ilies et al, 2018 | Multiple-gate | PDAC *vs* benign disease | 14/17 | IPMN, CP | Plasma | TIMP-1 | - | 720 ng/mL | 11 | 2 | 3 | 15 |  |
|  |  |  |  |  |  | CA19 | - | - | - | - | - | - |  |
| Jenkinson et al, 2015 | Multiple-gate | PDAC *vs* benign disease | 40/20 | CP, benign biliary obstruction | Serum | TIMP-1 | - | 320 ng/mL | 28 | 10 | 12 | 10 |  |
|  |  |  |  |  |  | ICAM-1 | - | 530 ng/mL | 19 | 4 | 21 | 16 |  |
|  |  |  |  |  |  | CA19 | - | - | - | - | - | - |  |
|  |  | PDAC *vs* healthy controls | 40/20 |  | Serum | TIMP-1 | - | 320 ng/mL | 28 | 1 | 12 | 19 |  |
|  |  |  |  |  |  | ICAM-1 | - | 260 ng/mL | 30 | 2 | 10 | 18 |  |
|  |  |  |  |  |  | CA19 | - | - | - | - | - | - |  |
| Kendrick et al, 2013 | Multiple-gate | PDAC *vs* benign disease | 84/40 | CP | Serum | IGFBP2 | 0.69 (0.59-0.79) | 480 pg/mL | 27 | 2 | 57 | 38 |  |
|  |  |  |  |  |  | CA19 | 0.91 (0.85-0.97) | 37 U/mL | 71 | 5 | 13 | 35 |  |
|  |  | PDAC *vs* healthy controls | 84/84 |  | Serum | IGFBP2 | 0.63 (0.54-0.71) | 770 pg/mL | 27 | 19 | 57 | 65 |  |
|  |  |  |  |  |  | CA19 | 0.93 (0.88-0.97) | 37 U/mL | 71 | 9 | 13 | 75 |  |
| Kim et al.,  2017 | Multiple-gate | PDAC *vs* benign disease | 197/115 | IPMN, CP | Plasma | THBS2 | 0.78 (0.74-0.82) | - | 168 | 59 | 29 | 56 |  |
|  |  |  |  |  |  | CA19-9 | 0.88 (0.86-0.91) | 55 U/mL | 175 | 57 | 22 | 58 |  |
|  |  | PDAC *vs* healthy controls | 197/140 |  | Plasma | THBS2 | 0.88 (0.85-0.90) | 42 ng/mL | 102 | 1 | 95 | 139 |  |
|  |  |  |  |  |  | CA19-9 | 0.88 (0.86-0.91) | 55 U/mL | 153 | 2 | 44 | 138 |  |
| Kim et al,  2021 | Multiple-gate | PDAC *vs* benign disease | 75/47 | IPMN, NET,  SPN, pancreatitis | Plasma | ICAM-1 | 0.68 (0.57-0.78) | - | - | - | - | - |  |
|  |  |  |  |  |  | IGFBP2 | 0.55 (0.44-0.66) | - | - | - | - | - |  |
|  |  |  |  |  |  | CA19-9 | 0.77 (0.69-0.86) | - | 46 | 2 | 29 | 45 |  |
| Koopman et al, 2004a | Multiple-gate | PDAC *vs* healthy controls | 50/22 |  | Serum | OPN | - | 334 ng/mL | 40 | 1 | 10 | 21 |  |
|  |  |  |  |  |  | CA19 | - | 70 U/mL | 30 | - | 18 | - |  |
| Koopman et al, 2004b | Multiple-gate | PDAC *vs* benign disease | 80/216 | CP, IPMN, NET, cystadenoma | Serum | MIC-1 | 0.81 (0.75-0.86) | 1070 pg/mL | 57 | 47 | 23 | 169 |  |
|  |  |  |  |  |  | CA19 | 0.77 (0.69-0.83) | 70 U/mL | 47 | 26 | 33 | 190 |  |
|  |  | PDAC *vs* healthy controls | 80/97 |  | Serum | MIC-1 | - | 1070 pg/mL | 57 | 4 | 23 | 93 |  |
|  |  |  |  |  |  | CA19 | - | - | - | - | - | - |  |
| Koopman et al, 2006 | Multiple-gate | PDAC *vs* benign disease | 50/50 | CP | Serum | MIC-1 | 0.81 (0.68-0.92) | 1583 pg/mL | 45 | 28 | 5 | 22 |  |
|  |  |  |  |  |  | TIMP-1 | 0.66 (0.55-0.77) | 1564 ng/mL | 25 | 14 | 25 | 36 |  |
|  |  |  |  |  |  | OPN | 0.72 (0.62-0.82) | 747 ng/mL | 17 | 4 | 33 | 46 |  |
|  |  |  |  |  |  | CA19 | 0.74 (0.64-0.84) | 37 U/mL | 31 | 7 | 19 | 43 |  |
|  |  | PDAC *vs* healthy controls | 50/50 |  | Serum | MIC-1 | 0.99 (0.97-1.00) | 1583 pg/mL | 45 | 3 | 5 | 47 |  |
|  |  |  |  |  |  | TIMP-1 | 0.85 (0.77-0.93) | 1564 ng/mL | 25 | 2 | 25 | 48 |  |
|  |  |  |  |  |  | OPN | 0.86 (0.79-0.93) | 747 ng/mL | 17 | 3 | 33 | 47 |  |
|  |  |  |  |  |  | CA19 | 0.78 (0.69-0.87) | 37 U/mL | 31 | 10 | 19 | 40 |  |
| Le Large et al, 2020 | Multiple-gate | PDAC *vs* benign disease | 82/31 | CP, cholangitis, gallstones | Plasma | THBS2 | 0.60 (0.48-0.72) | 42 ng/mL | 45 | 11 | 37 | 19 |  |
|  |  |  |  |  |  | CA19 | 0.78 (0.68-0.87) | 37 U/mL | 48 | 3 | 34 | 27 |  |
|  |  | PDAC *vs* healthy controls | 82/50 |  | Plasma | THBS2 | 0.82 (0.75-0.89) | 42 ng/mL | 45 | 2 | 37 | 48 |  |
|  |  |  |  |  |  | CA19 | 0.87 (0.81-0.93) | 37 U/mL | 48 | 0 | 34 | 50 |  |
| Mohamed et al, 2015 | Multiple-gate | PDAC *vs* healthy controls | 50/20 |  | Serum | MIC-1 | 0.92 (0.84-0.99) | 2070 pg/mL | 47 | 11 | 3 | 9 |  |
|  |  |  |  |  |  | CA19 | 0.90 (0.82-0.99) | 55 U/mL | 41 | 7 | 9 | 13 |  |
| Özkan et al, 2011 | Multiple-gate | PDAC *vs* benign disease | 56/31 | AP, CP | Serum | MIC-1 | 0.72 (0.62-0.82) | 2686 pg/mL | 35 | 6 | 21 | 25 |  |
|  |  |  |  |  |  | CA19 | 0.86 (0.77-0.92) | 34 U/mL | 45 | 9 | 11 | 22 |  |
|  |  | PDAC *vs* healthy controls | 56/33 |  | Serum | MIC-1 | 0.88 (0.78-0.94) | 1259 pg/mL | 45 | 9 | 11 | 24 |  |
|  |  |  |  |  |  | CA19 | 0.93 (0.85-0.97) | 34 U/mL | 45 | 1 | 11 | 32 |  |
| Pan et al,  2011 | Multiple-gate | PDAC *vs* healthy control | 45/41 |  | Plasma | TIMP-1 | 0.83 (0.74-0.92) | - | 24 | 1 | 21 | 40 |  |
|  |  |  |  |  |  | ICAM-1 | 0.83 (0.74-0.92) | - | 29 | 1 | 16 | 40 |  |
|  |  |  |  |  |  | CA19 | 0.58 (0.46-0.70) | - | 43 | 40 | 2 | 1 |  |
| Pan et al,  2012 | Multiple-gate | PDAC *vs* benign disease | 20/20 | CP | Plasma | TIMP-1 | - | 570 ng/mL | 16 | 5 | 4 | 15 |  |
|  |  |  |  |  |  | CA19 | - | - | - | - | - | - |  |
|  |  | PDAC *vs* healthy controls | 20/20 |  | Plasma | TIMP-1 | - | 570 ng/mL | 16 | 2 | 4 | 18 |  |
|  |  |  |  |  |  | CA19 | - | - | - | - | - | - |  |
| Poruk et al, 2013 | Multiple-gate | PDAC *vs* benign disease | 86/48 | CP | Serum | TIMP-1 | 0.78 (0.70-0.86) | 120 ng/mL | 83 | 47 | 3 | 1 |  |
|  |  |  |  |  |  | OPN | 0.71 (0.62-0.80) | 100 ng/mL | 19 | 3 | 67 | 45 |  |
|  |  |  |  |  |  | CA19 | 0.91 (0.85-0.96) | 37 U/mL | 72 | 7 | 14 | 41 |  |
|  |  | PDAC *vs* healthy controls | 86/86 |  | Serum | TIMP-1 | 0.77 (0.70-0.84) | 100 ng/mL | 85 | 83 | 1 | 3 |  |
|  |  |  |  |  |  | OPN | 0.75 (0.67-0.82) | 100 ng/mL | 19 | 3 | 67 | 83 |  |
|  |  |  |  |  |  | CA19 | 0.93 (0.88-0.97) | 37 U/mL | 72 | 9 | 14 | 77 |  |
| Resovi et al, 2018 | Multiple-gate | PDAC *vs* benign disease | 131/30 | CP | Plasma | THBS2 | 0.57 (0.46-0.68) | 360 ng/mL | 7 | 2 | 124 | 28 |  |
|  |  |  |  |  |  | TIMP-1 | 0.66 (0.55-0.77) | 120 ng/mL | 25 | 2 | 106 | 28 |  |
|  |  |  |  |  |  | ICAM-1 | 0.57 (0.46-0.68) | 460 ng/mL | 25 | 3 | 106 | 27 |  |
|  |  |  |  |  |  | IGFBP2 | 0.59 (0.48-0.70) | - | 30 | 2 | 101 | 28 |  |
|  |  |  |  |  |  | CA19 | 0.83 (0.75-0.91) | - | 108 | 6 | 23 | 24 |  |
|  |  | PDAC *vs* healthy controls | 131/131 |  | Plasma | THBS2 | 0.78 (0.70-0.86) | - | 86 | 25 | 45 | 106 |  |
|  |  |  |  |  |  | TIMP-1 | 0.82 (0.77-0.87) | 100 ng/mL | 45 | 0 | 86 | 131 |  |
|  |  |  |  |  |  | ICAM-1 | 0.77 (0.71-0.84) | 460 ng/mL | 25 | 2 | 106 | 129 |  |
|  |  |  |  |  |  | IGFBP2 | 0.82 (0.77-0.87) | - | 30 | 0 | 101 | 131 |  |
|  |  |  |  |  |  | CA19 | 0.87 (0.81-0.93) | - | 107 | 24 | 24 | 107 |  |
| Rychlíková et al, 2016 | Multiple-gate | PDAC *vs* benign disease | 62/71 | CP | Plasma | OPN (62/71) | 0.75 (0.66-0.84) | 100 ng/mL | 40 | 16 | 22 | 55 |  |
|  |  |  |  |  |  | CA19 (61/70) | 0.86 (0.79-0.93) | 37 U/mL | 46 | 10 | 15 | 60 |  |
|  |  | PDAC *vs* healthy controls | 62/48 |  | Plasma | OPN (62/48) | 0.88 (0.82-0.95) | 80 ng/mL | 48 | 3 | 14 | 45 |  |
|  |  |  |  |  |  | CA19 (61/47) | 0.91 (0.84-0.97) | 37 U/mL | 46 | 0 | 15 | 47 |  |
| Simpson et al, 2018 | Multiple-gate | PDAC *vs* benign disease | 30/100 | IPMN | Plasma & serum | THBS2 (plasma) | - | 34 ng/mL | 13 | 8 | 17 | 92 |  |
|  |  |  |  |  |  | CA19 (serum) | - | - | - | - | - | - |  |
| Song et al,  2019 | Multiple-gate | PDAC *vs* benign disease | 188/131 | CP, IPMN | Serum | MIC-1 | 0.69 (0.64-0.74) | - | 60 | 20 | 128 | 111 |  |
|  |  |  |  |  |  | OPN | 0.65 (0.59-0.70) | - | 165 | 89 | 23 | 42 |  |
|  |  |  |  |  |  | CA19-9 | 0.82 (0.77-0.86) | - | 69 | 9 | 119 | 122 |  |
|  |  | PDAC *vs* healthy controls | 188/89 |  | Serum | MIC-1 | 0.97 (0.95-0.99) | - | 175 | 11 | 13 | 78 |  |
|  |  |  |  |  |  | OPN | 0.90 (0.86-0.94) | - | 173 | 23 | 15 | 66 |  |
|  |  |  |  |  |  | CA19-9 | 0.93 (0.90-0.96) | - | 163 | 29 | 25 | 60 |  |
| Wang et al, 2014 | Multiple-gate | PDAC *vs* benign disease | 807/50 | CP | Serum | MIC-1 | 0.59 (0.51-0.67) | - | - | - | - | - |  |
|  |  |  |  |  |  | CA19 | 0.68 (0.60-0.76) | - | - | - | - | - |  |
|  |  | PDAC *vs* healthy controls | 807/665 |  | Serum | MIC-1 | 0.94 (0.92-0.95) | 1000 pg/mL | 531 | 24 | 276 | 641 |  |
|  |  |  |  |  |  | CA19 | 0.80 (0.78-0.82) | - | 430 | 24 | 377 | 641 |  |
| Yoneyama et al, 2016 | Multiple-gate | PDAC *vs* healthy controls | 38/65 |  | Plasma | IGFBP2 | 0.71 (0.60-0.81) | 20 pmol/L | 26 | 21 | 12 | 44 |  |
|  |  |  |  |  |  | CA19 | 0.84 (0.75-0.93) | 37 U/mL | 23 | 9 | 15 | 56 |  |
| Zhou et al, 1998 | Multiple-gate | PDAC *vs* healthy controls | 85/98 |  | Serum | TIMP-1 | - | 1550 ng/mL | 43 | 2 | 42 | 96 |  |
|  |  |  |  |  |  | CA19 | - | 137 U/mL | 49 | 1 | 39 | 97 |  |

AP, acute pancreatitis; AIP, auto-immune pancreatitis; CP, chronic pancreatitis; IPMN, intraductal papillary mucinous neoplasms; NET, neuroendocrine tumor; SPN, solid-pseudopapillary neoplasm.

| **Supplementary table 3. Bayesian bivariate meta-regression of THBS2 *vs* CA19-9 for PDAC *vs* benign disease.** | | |
| --- | --- | --- |
| **Estimate** | **THBS2 (42 ng/mL)** | **CA19-9 (37 U/mL)** |
| Sensitivity (95% CrI) | 0.66 (0.49 to 0.80) | 0.67 (0.50 to 0.80) |
| Specificity (95% CrI) | 0.63 (0.37 to 0.83) | 0.87 (0.70 to 0.95) |
| Difference in sensitivity (95% CrI) | 0.01 (-0.22 to 0.22) | |
| Difference in specificity (95% CrI) | 0.23 (-0.02 to 0.51) | |
| CrI denotes credible interval. | | |

| **Supplementary table 4. Bayesian bivariate meta-regression of PDAC *vs* benign disease compared with PDAC *vs* healthy controls for THBS2.** | | |
| --- | --- | --- |
| **Estimate** | **PDAC *vs* benign disease** | **PDAC *vs* healthy controls** |
| Sensitivity (95% CrI) | 0.67 (0.45 to 0.82) | 0.68 (0.48 to 0.84) |
| Specificity (95% CrI) | 0.64 (0.25 to 0.91) | 0.90 (0.62 to 0.98) |
| Difference in sensitivity (95% CrI) | 0.02 (-0.24 to 0.28) | |
| Difference in specificity (95% CrI) | 0.25 (-0.12 to 0.66) | |
| CrI, credible interval. | | |

| **Supplementary table 5. Pairwise comparisons between protein biomarkers for PDAC *vs* healthy controls.** | | | | | | | |
| --- | --- | --- | --- | --- | --- | --- | --- |
|  | MIC-1 | THBS2 | TIMP-1 | OPN | ICAM-1 | IGFBP2 |  |
| MIC-1 | - | 1.84  (0.41-8.41) | 1.48  (0.63-3.49) | **4.22^a^**  **(1.62-11.0)** | 2.72  (0.63-11.7) | **5.75^a^**  **(1.30-25.3)** |  |
| THBS2 | 0.54  (0.12-2.46) | - | **0.15^a^**  **(0.03-0.85)** | 1.21  (0.29-5.10) | 0.98  (0.24-3.97) | 0.89  (0.14-5.81) |  |
| TIMP-1 | 0.68  (0.29-1.58) | **6.69^a^**  **(1.17-38.1)** | - | 2.14  (0.77-5.93) | 2.32  (0.73-7.24) | **3.60^a^**  **(1.03-12.6)** |  |
| OPN | **0.24^a^**  **(0.09-0.62)** | 0.83  (0.20-3.49) | 0.47  (0.17-1.30) | - | 1.16  (0.25-5.47) | 2.51  (0.50-12.4) |  |
| ICAM-1 | 0.37  (0.09-1.58) | 1.02  (0.25-4.18) | 0.43  (0.14-1.36) | 0.86  (0.18-4.06) | - | 0.96  (0.11-8.85) |  |
| IGFBP2 | **0.17^a^**  **(0.04-0.77)** | 1.13  (0.17-7.39) | **0.28^a^**  **(0.08-0.97)** | 0.40  (0.08-1.99) | 1.04  (0.11-9.49) | - |  |
| Relative diagnostic accuracy (relative diagnostic odds ratio) of protein biomarkers for PDAC *vs* healthy controls, based on mostly indirect comparisons. Comparisons are reported as “row *vs* column” and are displayed twice. Confidence intervals were not adjusted for multiplicity and should be interpreted with caution.  a: *P*<0.05. Likelihood ratio test. | | | | | | | |

| **Supplementary table 6. Pairwise comparisons between protein biomarkers for PDAC *vs* benign disease.** | | | | | | |
| --- | --- | --- | --- | --- | --- | --- |
|  | MIC-1 | THBS2 | TIMP-1 | OPN | ICAM-1 | IGFBP2 |
| MIC-1 | - | 1.57  (0.59-4.14) | 1.13  (0.44-2.89) | 0.78  (0.29-2.08) | 1.39  (0.35-5.53) | 0.96  (0.28-3.39) |
| THBS2 | 0.64  (0.24-1.68) | - | 0.50  (0.15-1.62) | 0.98  (0.28-3.35) | 0.64  (0.12-3.35) | 0.54  (0.12-2.48) |
| TIMP-1 | 0.89  (0.35-2.27) | 2.01  (0.62-6.49) | - | 1.05  (0.21-5.16) | 1.13  (0.31-4.18) | **0.22^a^**  **(0.06-0.75)** |
| OPN | 1.28  (0.48-3.42) | 1.02  (0.30-3.53) | 0.95  (0.19-4.71) | - | - | - |
| ICAM-1 | 0.72  (0.18-2.86) | 1.55  (0.30-8.00) | 0.89  (0.24-3.25) | - | - | - |
| IGFBP2 | 1.04  (0.30-3.63) | 1.86  (0.40-8.58) | **4.57^a^**  **(1.34-15.6)** | - | - | - |
| Relative diagnostic accuracy of protein biomarkers for PDAC *vs* benign disease, based on mostly indirect comparisons. Pairwise comparisons are reported as “row *vs* column” and are displayed twice. Confidence intervals were not adjusted for multiplicity and should be interpreted with caution.  a: *P*<.05. Likelihood ratio test. | | | | | | |

| **Supplementary table 7.** **Summary of meta-analysis and meta-regression results**. | | | | |
| --- | --- | --- | --- | --- |
| **Biomarker** | **AUC**  **(95% CI)** | **AUC CA19-9**  **(95% CI)** | **rDOR (95% CI)**  ***vs* CA19-9** | **rDOR (95% CI) *vs***  **PDAC *vs* HC** |
| **PDAC *vs* healthy controls** | | | | |
| MIC-1 | 0.95  (0.86-0.98) | 0.87  (0.74-0.94) | 2.32  (1.28-4.14) | - |
| THBS2 | 0.83  (0.74-0.90) | 0.88  (0.84-0.91) | 0.37  (0.10-1.46) | - |
| TIMP-1 | 0.82  (0.76-0.86) | 0.88  (0.75-0.95) | 0.33  (0.19-0.58) | - |
| OPN | 0.82  (0.67-0.91) | 0.90  (0.81-0.95) | 0.33  (0.17-0.64) | - |
| ICAM-1 | 0.78  (0.65-0.87) | - | 0.80  (0.18-3.63) | - |
| IGFBP2 | 0.75  (0.58-0.86) | 0.88  (0.81-0.93) | 0.16  (0.04-0.55) | - |
| **PDAC *vs* benign disease** | | | | |
| MIC-1 | 0.75  (0.69-0.81) | 0.75  (0.61-0.86) | 0.48  (0.22-1.02) | 0.14  (0.04-0.48) |
| THBS2 | 0.67  (0.54-0.79) | 0.83  (0.76-0.89) | 0.22  (0.11-0.46) | 0.12  (0.06-0.27) |
| TIMP-1 | 0.72  (0.62-0.80) | 0.83  (0.73-0.90) | 0.12  (0.06-0.26) | 0.13  (0.06-0.30) |
| OPN | 0.69  (0.61-0.77) | 0.83  (0.69-0.92) | 0.24  (0.06-0.89) | 0.17  (0.08-0.38) |
| ICAM-1 | - | 0.80  (0.66-0.89) | 0.11  (0.01-1.15) | 0.25  (0.04-1.72) |
| IGFBP2 | 0.62  (0.52-0.70) | 0.83  (0.72-0.90) | 0.22  (0.04-1.28) | 0.41  (0.08-2.12) |

| **Supplementary table 8. Clinical implications of using blood-based protein biomarkers as a triage test.** | | | | | | | | |
| --- | --- | --- | --- | --- | --- | --- | --- | --- |
| **Biomarker** | **Prevalence of PDAC** | **If test positive** | | |  | **If test negative** | | |
|  |  | No PDAC but given diagnostic intervention | PDAC and diagnostic intervention performed | Proportion of diagnostic interventions performed correctly |  | Diagnostic intervention not performed | PDAC cases missed | Proportion of diagnostic interventions avoided correctly |
| **MIC-1** | 10% | 198 | 62 | 23.8% |  | 740 | 38 | 94.9% |
|  | 30% | 154 | 186 | 54.7% |  | 660 | 114 | 82.7% |
|  | 50% | 110 | 310 | 73.8% |  | 580 | 190 | 67.2% |
| **THBS2** | 10% | 198 | 52 | 20.8% |  | 750 | 48 | 93.6% |
|  | 30% | 154 | 156 | 50.3% |  | 690 | 146 | 78.8% |
|  | 50% | 110 | 260 | 70.3% |  | 630 | 240 | 61.9% |
| **TIMP-1** | 10% | 225 | 68 | 23.2% |  | 707 | 32 | 95.5% |
|  | 30% | 175 | 204 | 53.8% |  | 621 | 96 | 84.5% |
|  | 50% | 125 | 340 | 73.1% |  | 535 | 160 | 70.0% |
| **ICAM-1** | 10% | 198 | 53 | 21.1% |  | 749 | 47 | 93.7% |
|  | 30% | 140 | 159 | 53.2% |  | 701 | 141 | 79.9% |
|  | 50% | 100 | 265 | 72.6% |  | 635 | 235 | 63.0% |
| Clinical implications of using a single protein biomarker in the clinical management of a hypothetical cohort of 1000 patients suspected of PDAC, assuming (1) a cohort consisting solely of patients with PDAC and patients with benign disease, (2) a pre-test probability of 10%, 30%, or 50%, (3) no correlation between the prevalence of PDAC and the sensitivity and specificity of the protein biomarker; (4) a diagnostic intervention, e.g., EUS, CT, MRI, or biopsy, for all patients with a positive test, (5) no diagnostic intervention for patients with a negative test. Sensitivity was estimated directly from the HSROC model results for the median observed specificity of studies assessing a particular biomarker. As most included studies did not use a single-gate design, a reliable estimate for the prevalence of PDAC in patients with suspected PDAC could not be determined from these studies and results are reported for a range of pre-test probabilities. | | | | | | | | |

**Appendix 1 – Database search strategies**

**PubMed Session Results (June 15, 2022)**

| Search | Query | Items found |
| --- | --- | --- |
| #5 | #1 AND #2 AND #3 AND #4 | 4,199 |
| #4 | "Sensitivity and Specificity"[Mesh] OR "Area Under Curve"[Mesh] OR "diagnostic"[tiab] OR "diagnosis"[tiab] OR "diagnoses"[tiab] OR "diagnosing"[tiab] OR "diagnose"[tiab] OR "detection"[tiab] OR "detect"[tiab] OR "detecting"[tiab] OR "receiver operati*"[tiab] OR "relative operati*"[tiab] OR "area under the curve*"[tiab] OR "areas under the curve*"[tiab] OR "sensitiv*"[tiab] OR "specificit*"[tiab] OR "roc curve*"[tiab] OR "roc analys*"[tiab] | 5,161,787 |
| #3 | "Carcinoma, Pancreatic Ductal"[Mesh] OR "Cholangiocarcinoma"[Mesh:NoExp] OR "cholangiocarcinoma*"[tiab] OR "cholangio-carcinoma*"[tiab] OR "Duodenal Neoplasms"[Mesh] OR "Duodenum"[Mesh] OR "duodenum"[tiab] OR "duodenal"[tiab] OR "Ampulla of Vater"[Mesh] OR "hepatopancreatic ampulla"[tiab] OR "Ampulla of Vater"[tiab] OR "Vater Ampulla"[tiab] OR "Ampulla Vater*"[tiab] OR "Vater's Ampulla"[tiab] OR "periampulla*"[tiab] OR "peri ampulla*"[tiab] OR (("ductal"[tiab] OR "duct-cell"[tiab]) AND ("pancreas*"[tiab] OR "pancreat*"[tiab])) | 151,524 |
| #2 | "Adenocarcinoma"[Mesh] OR "malignant adenoma*"[tiab] OR "adenocarcinoma*"[tiab] OR "adeno carcinoma*"[tiab] OR "glandular carcinoma*"[tiab] | 489,725 |
| #1 | "Proto-Oncogene Proteins p21(ras)"[Mesh] OR "KRAS protein, human"[Supplementary Concept] OR "Methylation"[Mesh] OR "Extracellular Vesicles"[Mesh] OR "Liquid Biopsy"[Mesh] OR "Biomarkers"[Mesh] OR "biomarker*"[tiab] OR "biologic marker*"[tiab] OR "biological marker*"[tiab] OR "serum marker*"[tiab] OR "biochemical marker*"[tiab] OR "diagnostic marker*"[tiab] OR "blood test*"[tiab] OR "tumor marker*"[tiab] OR "tumour marker*"[tiab] OR "liquid biops*"[tiab] OR "fluid biops*"[tiab] OR "KRAS"[tiab] OR "K-ras*"[tiab] OR "Ki-ras*"[tiab] OR "kirsten-ras"[tiab] OR "Kirsten rat*"[tiab] OR "Ca19-9"[tiab] OR "cancer antigen 19-9"[tiab] OR "Exosome*"[tiab] OR "extracellular vesicle*"[tiab] OR "Thrombocyt*"[tiab] OR "platelet*"[tiab] OR "TEP"[tiab] OR "TEPS"[tiab] OR "circulating tumor DNA"[tiab] OR "circulating tumour DNA"[tiab] OR "CT-dna"[tiab] OR "CTdna"[tiab] OR "circulating tumor cell*"[tiab] OR "circulating tumour cell*"[tiab] OR "cftDNA"[tiab] OR "cft-DNA"[tiab] OR "circulating free tumor DNA"[tiab] OR "circulating free tumour DNA"[tiab] OR "circulating cell-free tumor DNA"[tiab] OR "circulating cell-free tumour DNA"[tiab] OR "circulating nucleic acids"[tiab] OR "circulating cell-free nucleic acid*"[tiab] OR "cell-free circulating nucleic acid*"[tiab] OR "plasma DNA"[tiab] OR "circulating DNA"[tiab] OR "circulating free DNA"[tiab] OR "cf-NA*"[tiab] OR "cfNA*"[tiab] OR "cfCNA*"[tiab] OR "cf-CNA*"[tiab] OR "circulating cell-free nucleic acid*"[tiab] OR "cell-free circulating nucleic acid*"[tiab] OR "cf-mtDNA"[tiab] OR "cfmtDNA"[tiab] OR "ccf-mtDNA"[tiab] OR "cell-free mitochondrial DNA"[tiab] OR "circulating mitochondrial DNA"[tiab] OR "lnc-RNA*"[tiab] OR "ncRNA*"[tiab] OR "nc-RNA*"[tiab] OR "noncoding RNA*"[tiab] OR "non-coding RNA*"[tiab] OR "cfDNA"[tiab] OR "cf-DNA"[tiab] OR "cell-free DNA"[tiab] OR "cell-free circulating DNA"[tiab] OR "miRNA*"[tiab] OR "mi-RNA*"[tiab] OR "micro RNA*"[tiab] OR "methylation"[tiab] | 1,641,209 |

**Embase Session Results (June 15, 2022)**

| Search | Query | Items found |
| --- | --- | --- |
| #5 | #1 AND #2 AND #3 AND #4 | 4,333 |
| #4 | 'sensitivity and specificity'/exp OR 'area under the curve'/exp OR 'receiver operating characteristic'/exp OR diagnostic:ab,ti,kw OR diagnosis:ab,ti,kw OR diagnoses:ab,ti,kw OR diagnosing:ab,ti,kw OR diagnose:ab,ti,kw OR detection:ab,ti,kw OR detect:ab,ti,kw OR detecting:ab,ti,kw OR 'receiver operati*':ab,ti,kw OR 'relative operati*':ab,ti,kw OR 'area under the curve*':ab,ti,kw OR 'areas under the curve*':ab,ti,kw OR sensitiv*:ab,ti,kw OR specificit*:ab,ti,kw OR 'roc curve*':ab,ti,kw OR 'roc analys*':ab,ti,kw | 6,723,946 |
| #3 | 'duodenum tumor'/exp OR 'duodenum'/exp OR 'ampulla of Vater'/exp OR cholangiocarcinoma*:ab,ti,kw OR 'cholangio-carcinoma*':ab,ti,kw OR duodenum:ab,ti,kw OR duodenal:ab,ti,kw OR 'hepatopancreatic ampulla':ab,ti,kw OR 'Ampulla of Vater':ab,ti,kw OR 'Vater Ampulla':ab,ti,kw OR 'Ampulla Vater*':ab,ti,kw OR 'Vater s Ampulla':ab,ti,kw OR ((ductal:ab,ti,kw OR 'duct-cell':ab,ti,kw) AND (pancreas*:ab,ti,kw OR pancreat*:ab,ti,kw)) | 196,023 |
| #2 | 'adenocarcinoma'/exp OR 'malignant adenoma*':ab,ti,kw OR adenocarcinoma*:ab,ti,kw OR 'adeno carcinoma*':ab,ti,kw OR 'glandular carcinoma*':ab,ti,kw OR periampulla*:ab,ti,kw OR ampullar*:ab,ti,kw OR 'peri ampulla*':ab,ti,kw | 377,394 |
| #1 | 'protein p21'/exp OR 'kras protein human'/exp OR 'methylation'/exp OR 'exosome'/exp OR 'liquid biopsy'/exp OR 'biological marker'/exp OR 'tumor marker'/exp OR biomarker*:ab,ti,kw OR 'biologic marker*':ab,ti,kw OR 'biological marker*':ab,ti,kw OR 'serum marker*':ab,ti,kw OR 'biochemical marker*':ab,ti,kw OR 'diagnostic marker*':ab,ti,kw OR 'blood test*':ab,ti,kw OR 'tumor marker*':ab,ti,kw OR 'tumour marker*':ab,ti,kw OR 'liquid biops*':ab,ti,kw OR 'fluid biops*':ab,ti,kw OR KRAS:ab,ti,kw OR 'K-ras*':ab,ti,kw OR 'Ki-ras*':ab,ti,kw OR 'kirsten-ras':ab,ti,kw OR 'Kirsten rat*':ab,ti,kw OR 'Ca19-9':ab,ti,kw OR 'cancer antigen 19-9':ab,ti,kw OR Exosome*:ab,ti,kw OR 'extracellular vesicle*':ab,ti,kw OR Thrombocyt*:ab,ti,kw OR platelet*:ab,ti,kw OR TEP:ab,ti,kw OR TEPS:ab,ti,kw OR 'circulating tumor DNA':ab,ti,kw OR 'circulating tumour DNA':ab,ti,kw OR 'CT-dna':ab,ti,kw OR CTdna:ab,ti,kw OR 'circulating tumor cell*':ab,ti,kw OR 'circulating tumour cell*':ab,ti,kw OR cftDNA:ab,ti,kw OR 'cft-DNA':ab,ti,kw OR 'circulating free tumor DNA':ab,ti,kw OR 'circulating free tumour DNA':ab,ti,kw OR 'circulating cell-free tumor DNA':ab,ti,kw OR 'circulating cell-free tumour DNA':ab,ti,kw OR 'circulating nucleic acids':ab,ti,kw OR 'circulating cell-free nucleic acid*':ab,ti,kw OR 'cell-free circulating nucleic acid*':ab,ti,kw OR 'plasma DNA':ab,ti,kw OR 'circulating DNA':ab,ti,kw OR 'circulating free DNA':ab,ti,kw OR 'cf-NA*':ab,ti,kw OR cfNA*:ab,ti,kw OR cfCNA*:ab,ti,kw OR 'cf-CNA*':ab,ti,kw OR 'circulating cell-free nucleic acid*':ab,ti,kw OR 'cell-free circulating nucleic acid*':ab,ti,kw OR 'cf-mtDNA':ab,ti,kw OR cfmtDNA:ab,ti,kw OR 'ccf-mtDNA':ab,ti,kw OR 'cell-free mitochondrial DNA':ab,ti,kw OR 'circulating mitochondrial DNA':ab,ti,kw OR 'lnc-RNA*':ab,ti,kw OR ncRNA*:ab,ti,kw OR 'nc-RNA*':ab,ti,kw OR 'noncoding RNA*':ab,ti,kw OR 'non-coding RNA*':ab,ti,kw OR cfDNA:ab,ti,kw OR 'cf-DNA':ab,ti,kw OR 'cell-free DNA':ab,ti,kw OR 'cell-free circulating DNA':ab,ti,kw OR miRNA*:ab,ti,kw OR 'mi-RNA*':ab,ti,kw OR 'micro RNA*':ab,ti,kw OR methylation:ab,ti,kw | 1,819,699 |

**Wiley / Cochrane Library Session Results (June 15, 2022)**

| Search | Query | Items found |
| --- | --- | --- |
| #5 | #1 AND #2 AND #3 AND #4 | 93 |
| #4 | (diagnostic OR diagnosis OR diagnoses OR diagnosing OR diagnose OR detection OR detect OR detecting OR (receiver NEXT operati*) OR (relative NEXT operati*) OR (area NEXT under NEXT the NEXT curve*) OR (areas NEXT under NEXT the NEXT curve*) OR sensitiv* OR specificit* OR (roc NEXT curve*) OR (roc NEXT analys*)):ab,ti,kw | 323,628 |
| #3 | (cholangiocarcinoma* OR (cholangio NEXT carcinoma*) OR duodenum OR duodenal OR (hepatopancreatic NEXT ampulla) OR (Ampulla NEXT of NEXT Vater) OR (Vater NEXT Ampulla) OR (Ampulla NEXT Vater*) OR (Vater's NEXT Ampulla) OR ((ductal OR (duct NEXT cell)) AND (pancreas* OR pancreat*))):ab,ti,kw | 9,194 |
| #2 | ((malignant NEXT adenoma*) OR adenocarcinoma* OR (adeno NEXT carcinoma*) OR (glandular NEXT carcinoma*) OR periampulla* OR ampullar* OR (peri NEXT ampulla*)):ab,ti,kw | 12,190 |
| #1 | (biomarker* OR (biologic NEXT marker*) OR (biological NEXT marker*) OR (serum NEXT marker*) OR (biochemical NEXT marker*) OR (diagnostic NEXT marker*) OR (blood NEXT test*) OR (tumor NEXT marker*) OR (tumour NEXT marker*) OR (liquid NEXT biops*) OR (fluid NEXT biops*) OR KRAS OR (K NEXT ras*) OR (Ki NEXT ras*) OR (Kirsten NEXT ras) OR (Kirsten NEXT rat*) OR (Ca19 NEXT 9) OR (cancer NEXT antigen NEXT 19 NEXT 9) OR Exosome* OR (extracellular NEXT vesicle*) OR Thrombocyt* OR platelet* OR TEP OR TEPS OR (circulating NEXT tumor NEXT DNA) OR (circulating NEXT tumour NEXT DNA) OR (CT NEXT dna) OR CTdna OR (circulating NEXT tumor NEXT cell*) OR (circulating NEXT tumour NEXT cell*) OR cftDNA OR (cft NEXT DNA) OR (circulating NEXT free NEXT tumor NEXT DNA) OR (circulating NEXT free NEXT tumour NEXT DNA) OR (circulating NEXT cell NEXT free NEXT tumor NEXT DNA) OR (circulating NEXT cell NEXT free NEXT tumour NEXT DNA) OR (circulating NEXT nucleic NEXT acids) OR (circulating NEXT cell NEXT free NEXT nucleic NEXT acid*) OR (cell NEXT free NEXT circulating NEXT nucleic NEXT acid*) OR (plasma NEXT DNA) OR (circulating NEXT DNA) OR (circulating NEXT free NEXT DNA) OR (cf NEXT NA*) OR cfNA* OR cfCNA* OR (cf NEXT CNA*) OR (circulating NEXT cell NEXT free NEXT nucleic NEXT acid*) OR (cell NEXT free NEXT circulating NEXT nucleic NEXT acid*) OR (cf NEXT mtDNA) OR cfmtDNA OR (ccf NEXT mtDNA) OR (cell NEXT free NEXT mitochondrial NEXT DNA) OR (circulating NEXT mitochondrial NEXT DNA) OR (lnc NEXT RNA*) OR ncRNA* OR (nc NEXT RNA*) OR (noncoding NEXT RNA*) OR (non NEXT coding NEXT RNA*) OR cfDNA OR (cf NEXT DNA) OR (cell NEXT free NEXT DNA) OR (cell NEXT free NEXT circulating NEXT DNA) OR miRNA* OR (mi NEXT RNA*) OR (micro NEXT RNA*) OR methylation):ab,ti,kw | 99,943 |

**Web of Science (Core Collection) Session Results (June 15, 2022)**

| Search | Query | Items found |
| --- | --- | --- |
| #5 | #1 AND #2 AND #3 AND #4 | 3,374 |
| #4 | TS=(diagnostic OR diagnosis OR diagnoses OR diagnosing OR diagnose OR detection OR detect OR detecting OR "receiver operati*" OR "relative operati*" OR "area under the curve*" OR "areas under the curve*" OR sensitiv* OR specificit* OR "roc curve*" OR "roc analys*") | 7,776,702 |
| #3 | TS=(cholangiocarcinoma* OR "cholangio-carcinoma*" OR duodenum OR duodenal OR "hepatopancreatic ampulla" OR "Ampulla of Vater" OR "Vater Ampulla" OR "Ampulla Vater*" OR "Vater s Ampulla" OR ((ductal OR "duct-cell") AND (pancreas* OR pancreat*))) | 125,567 |
| #2 | TS=("malignant adenoma*" OR adenocarcinoma* OR "adeno carcinoma*" OR "glandular carcinoma*" OR periampulla* OR ampullar* OR "peri ampulla*") | 220,984 |
| #1 | TS=(biomarker* OR "biologic marker*" OR "biological marker*" OR "serum marker*" OR "biochemical marker*" OR "diagnostic marker*" OR "blood test*" OR "tumor marker*" OR "tumour marker*" OR "liquid biops*" OR "fluid biops*" OR KRAS OR "K-ras*" OR "Ki-ras*" OR "kirsten-ras" OR "Kirsten rat*" OR "Ca19-9" OR "cancer antigen 19-9" OR Exosome* OR "extracellular vesicle*" OR Thrombocyt* OR platelet* OR TEP OR TEPS OR "circulating tumor DNA" OR "circulating tumour DNA" OR "CT-dna" OR CTdna OR "circulating tumor cell*" OR "circulating tumour cell*" OR cftDNA OR "cft-DNA" OR "circulating free tumor DNA" OR "circulating free tumour DNA" OR "circulating cell-free tumor DNA" OR "circulating cell-free tumour DNA" OR "circulating nucleic acids" OR "circulating cell-free nucleic acid*" OR "cell-free circulating nucleic acid*" OR "plasma DNA" OR "circulating DNA" OR "circulating free DNA" OR "cf-NA*" OR cfNA* OR cfCNA* OR "cf-CNA*" OR "circulating cell-free nucleic acid*" OR "cell-free circulating nucleic acid*" OR "cf-mtDNA" OR cfmtDNA OR "ccf-mtDNA" OR "cell-free mitochondrial DNA" OR "circulating mitochondrial DNA" OR "lnc-RNA*" OR ncRNA* OR "nc-RNA*" OR "noncoding RNA*" OR "non-coding RNA*" OR cfDNA OR "cf-DNA" OR "cell-free DNA" OR "cell-free circulating DNA" OR miRNA* OR "mi-RNA*" OR "micro RNA*" OR methylation) | 1,245,496 |

**Appendix 2 – PRISMA-DTA for Abstracts checklist**

| **Section/topic** | **#** | **PRISMA-DTA for Abstracts Checklist item** | **Reported on page #** |
| --- | --- | --- | --- |
| **TITLE and PURPOSE** | | |  |
| Title | 1 | Identify the report as a systematic review (+/- meta-analysis) of diagnostic test accuracy (DTA) studies. | 1 |
| Objectives | 2 | Indicate the research question, including components such as participants, index test, and target conditions. | 1, 2 |
| **METHODS** | | |  |
| Eligibility criteria | 3 | Include study characteristics used as criteria for eligibility. | 3 |
| Information sources | 4 | List the key databases searched and the search dates. | 1, 3 |
| Risk of bias & applicability | 5 | Indicate the methods of assessing risk of bias and applicability. | 3 |
| Synthesis of results | A1 | Indicate the methods for the data synthesis. | 1, 3, 4 |
| **RESULTS** | | |  |
| Included studies | 6 | Indicate the number and type of included studies and the participants and relevant characteristics of the studies (including the reference standard). | 1, 3, 4 |
| Synthesis of results | 7 | Include the results for the analysis of diagnostic accuracy, preferably indicating the number of studies and participants. Describe test accuracy including variability; if meta-analysis was done, include summary results and confidence intervals. | 1,  Results |
| **DISCUSSION** | | |  |
| Strengths and limitations | 9 | Provide a brief summary of the strengths and limitations of the evidence | Discussion (word count limitations) |
| Interpretation | 10 | Provide a general interpretation of the results and the important implications. | 1 |
| **OTHER** | | |  |
| Funding | 11 | Indicate the primary source of funding for the review. | 1 |
| Registration | 12 | Provide the registration number and the registry name | N/A |

**Appendix 3 – PRISMA-DTA checklist**

| **Section/topic** | **#** | | **PRISMA-DTA Checklist Item** | | | **Reported on page #** |  |
| --- | --- | --- | --- | --- | --- | --- | --- |
| **TITLE / ABSTRACT** | | | | | |  |  |
| Title | 1 | | Identify the report as a systematic review (+/- meta-analysis) of diagnostic test accuracy (DTA) studies. | | | 1 |  |
| Abstract | 2 | | Abstract: See PRISMA-DTA for abstracts. | | | 1 |  |
| **INTRODUCTION** | | | | | |  |  |
| Rationale | 3 | | Describe the rationale for the review in the context of what is already known. | | | 2 |  |
| Clinical role of index test | D1 | | State the scientific and clinical background, including the intended use and clinical role of the index test, and if applicable, the rationale for minimally acceptable test accuracy (or minimum difference in accuracy for comparative design). | | | 2 |  |
| Objectives | 4 | | Provide an explicit statement of question(s) being addressed in terms of participants, index test(s), and target condition(s). | | | 1-3 |  |
| **METHODS** | | | | | |  |  |
| Protocol and registration | 5 | | Indicate if a review protocol exists, if and where it can be accessed (e.g., Web address), and, if available, provide registration information including registration number. | | | **None** |  |
| Eligibility criteria | 6 | | Specify study characteristics (participants, setting, index test(s), reference standard(s), target condition(s), and study design) and report characteristics (e.g., years considered, language, publication status) used as criteria for eligibility, giving rationale. | | | Table S1 |  |
| Information sources | 7 | | Describe all information sources (e.g., databases with dates of coverage, contact with study authors to identify additional studies) in the search and date last searched. | | | Figure 1 |  |
| Search | 8 | | Present full search strategies for all electronic databases and other sources searched, including any limits used, such that they could be repeated. | | | Appendix |  |
| Study selection | 9 | | State the process for selecting studies (i.e., screening, eligibility, included in systematic review, and, if applicable, included in the meta-analysis). | | | 2  Figure 1 |  |
| Data collection process | 10 | | Describe method of data extraction from reports (e.g., piloted forms, independently, in duplicate) and any processes for obtaining and confirming data from investigators. | | | 2 |  |
| Definitions for data extraction | 11 | | Provide definitions used in data extraction and classifications of target condition(s), index test(s), reference standard(s) and other characteristics (e.g. study design, clinical setting). | | | 2 |  |
| Risk of bias and applicability | 12 | | Describe methods used for assessing risk of bias in individual studies and concerns regarding the applicability to the review question. | | | 2 |  |
| Diagnostic accuracy measures | 13 | | State the principal diagnostic accuracy measure(s) reported (e.g. sensitivity, specificity) and state the unit of assessment (e.g. per-patient, per-lesion). | | | 2, 3 |  |
| Synthesis of results | 14 | | Describe methods of handling data, combining results and describing variability between studies. This could include, but is not limited to: a) handling of multiple definitions of target condition. b) handling of multiple thresholds of test positivity, c) handling multiple index test readers, d) handling of indeterminate test results, e) grouping and comparing tests, f) handling of different reference standards | | | 2-4 |  |
| **Section/topic** | | **#** | | **PRISMA-DTA Checklist Item** | **Reported on page #** | | |
| Meta-analysis | | D2 | | Report the statistical methods used for meta-analyses, if performed. | 3, 4 | | |
| Additional analyses | | 16 | | Describe methods of additional analyses (e.g., sensitivity or subgroup analyses, meta-regression), if done, indicating which were pre-specified. | 3, 4  Supplements | | |
| **RESULTS** | | | | |  | | |
| Study selection | | 17 | | Provide numbers of studies screened, assessed for eligibility, included in the review (and included in meta-analysis, if applicable) with reasons for exclusions at each stage, ideally with a flow diagram. | 4  Figure 1 | | |
| Study characteristics | | 18 | | For each included study provide citations and present key characteristics including: a) participant characteristics (presentation, prior testing), b) clinical setting, c) study design, d) target condition definition, e) index test, f) reference standard, g) sample size, h) funding sources | 4,  Table S2 | | |
| Risk of bias and applicability | | 19 | | Present evaluation of risk of bias and concerns regarding applicability for each study. | Figure S1 | | |
| Results of individual studies | | 20 | | For each analysis in each study (e.g. unique combination of index test, reference standard, and positivity threshold) report 2x2 data (TP, FP, FN, TN) with estimates of diagnostic accuracy and confidence intervals, ideally with a forest or receiver operator characteristic (ROC) plot. | Table S2 | | |
| Synthesis of results | | 21 | | Describe test accuracy, including variability; if meta-analysis was done, include results and confidence intervals. | 4-9 | | |
| Additional analysis | | 23 | | Give results of additional analyses, if done (e.g., sensitivity or subgroup analyses, meta-regression; analysis of index test: failure rates, proportion of inconclusive results, adverse events). | Figure S6, S7 | | |
| **DISCUSSION** | | | | |  | | |
| Summary of evidence | | 24 | | Summarize the main findings including the strength of evidence. | 9, 11 | | |
| Limitations | | 25 | | Discuss limitations from included studies (e.g. risk of bias and concerns regarding applicability) and from the review process (e.g. incomplete retrieval of identified research). | 9, 11 | | |
| Conclusions | | 26 | | Provide a general interpretation of the results in the context of other evidence. Discuss implications for future research and clinical practice (e.g. the intended use and clinical role of the index test). | 9, 11 | | |
| **FUNDING** | | | | |  | | |
| Funding | | 27 | | For the systematic review, describe the sources of funding and other support and the role of the funders. | 1, 12 | | |
