# Supplementary material for: Diagnostic accuracy and added value of blood-based protein biomarkers for pancreatic cancer: A meta-analysis of aggregate and individual participant data
Source: eClinicalMedicine. 2022 Nov 24;55:101747. doi: 10.1016/j.eclinm.2022.101747 (PMC9706531; doi:10.1016/j.eclinm.2022.101747)
Supplement: Supplementary Figures S1–S18, Tables S1–S8, and Appendices 1–3 Captions [file mmc2.docx]

**Supplementary Data – Captions**

Supplementary Figure 1: Quality assessment of studies included in the diagnostic

meta-analysis.

Supplementary Figure 2: Pooled AUC of protein biomarkers *vs* CA19-9 in direct,

head-to-head comparisons.

Supplementary Figure 3: AUC of blood-based proteins and CA19-9 as diagnostic

Biomarkers for PDAC *vs* healthy controls.

Supplementary Figure 4: AUC of blood-based proteins and CA19-9 as diagnostic

biomarkers for PDAC *vs* benign disease.

Supplementary Figure 5: Incremental diagnostic accuracy (∆AUC) from adding

blood-based proteins to CA19-9.

Supplementary Figure 6: Relative diagnostic accuracy of proteins and CA19-9, restricted to comparative studies.

Supplementary Figure 7: Relative diagnostic accuracy of protein biomarkers for PDAC *vs*

healthy controls and PDAC *vs* benign disease, restricted to

comparative studies.

Supplementary Figure 8: Incremental clinical value of blood-based proteins compared

with CA19-9 for PDAC *vs* benign disease, assuming different pre-test probabilities for PDAC.

Supplementary Figure 9: Discrimination, calibration, and clinical utility of THBS2 in Le

Large et al. (2020) for PDAC *vs* benign disease.

Supplementary Figure 10: Discrimination, calibration, and clinical utility of OPN in

Rychlíková et al. (2016) for PDAC *vs* benign disease.

Supplementary Figure 11: Discrimination, calibration, and clinical utility of OPN in Cohen

et al. (2017) for PDAC *vs* healthy controls.

Supplementary Figure 12: Discrimination, calibration, and clinical utility of OPN and

TIMP-1 in Poruk et al. (2013) for PDAC *vs* benign disease.

Supplementary Figure 13: Discrimination, calibration, and clinical utility of IGFBP2 in

Kendrick et al. (2013) for PDAC *vs* benign disease.

Supplementary Figure 14: Discrimination, calibration, and clinical utility of THBS2 in

Berger et al. (2019) for PDAC *vs* benign disease.

Supplementary Figure 15: Discrimination, calibration, and clinical utility of THBS2 in

Byrling et al. (2021) for PDAC *vs* benign disease.

Supplementary Figure 16: Discrimination and calibration of THBS2, TIMP-1, ICAM-1, and

IGFBP2 in Resovi et al. (2018) *for* PDAC vs benign disease.

Supplementary Figure 17: Non-linear relationships between CA19-9 and PDAC in

individual studies.

Supplementary Figure 18: Non-linear relationships between proteins and PDAC in

individual studies.

Supplementary Table 1: Eligibility criteria.

Supplementary Table 2: Characteristics of included studies.

Supplementary Table 3: Bayesian bivariate meta-regression of THBS2 *vs* CA19-9 for

PDAC *vs* benign disease.

Supplementary Table 4: Bayesian bivariate meta-regression of PDAC *vs* benign disease

compared with PDAC *vs* healthy controls for THBS2.

Supplementary Table 5: Pairwise comparisons between protein biomarkers for PDAC *vs*

healthy controls.

Supplementary Table 6: Pairwise comparisons between protein biomarkers for PDAC *vs*

benign disease.

Supplementary Table 7: Summary of meta-analysis and meta-regression results.

Supplementary Table 8: Clinical implications of using blood-based protein biomarkers

as a triage test.

Appendix 1: Database search strategies

Appendix 2: PRISMA-DTA for Abstracts checklist.

Appendix 3: PRISMA-DTA checklist.
